# Enhancer Hijacking Discovery in Acute Myeloid Leukemia by Pyjacker Identifies MNX1 Activation via Deletion 7q

**DOI:** 10.1158/2643-3230.BCD-24-0278

**Published:** 2025-03-31

**Authors:** Etienne Sollier, Anna Riedel, Umut H. Toprak, Justyna A. Wierzbinska, Dieter Weichenhan, Jan Philipp Schmid, Mariam Hakobyan, Aurore Touzart, Ekaterina Jahn, Binje Vick, Fiona Brown-Burke, Katherine Kelly, Simge Kelekçi, Anastasija Pejkovska, Ashish Goyal, Marion Bähr, Kersten Breuer, Mei-Ju May Chen, Maria Llamazares-Prada, Mark Hartmann, Maximilian Schönung, Nadia Correia, Andreas Trumpp, Yomn Abdullah, Ursula Klingmüller, Sadaf S. Mughal, Benedikt Brors, Frank Westermann, Elias Ulrich, Robert J. Autry, Matthias Schlesner, Sebastian Vosberg, Tobias Herold, Philipp A. Greif, Dietmar Pfeifer, Michael Lübbert, Thomas Fischer, Florian H. Heidel, Claudia Gebhard, Wencke Walter, Torsten Haferlach, Ann-Kathrin Eisfeld, Krzysztof Mrózek, Deedra Nicolet, Lars Bullinger, Leonie Smeenk, Claudia Erpelinck-Verschueren, Roger Mulet-Lazaro, Ruud Delwel, Aurélie Ernst, Michael Scherer, Pavlo Lutsik, Irmela Jeremias, Konstanze Döhner, Hartmut Döhner, Daniel B. Lipka, Christoph Plass

**Affiliations:** 1 Division of Cancer Epigenomics, German Cancer Research Center (DKFZ), Heidelberg, Germany.; 2 Division of Neuroblastoma Genomics, German Cancer Research Center (DKFZ), Heidelberg, Germany.; 3 Research Unit Apoptosis in Hematopoietic Stem Cells, Helmholtz Munich, German Research Center for Environmental Health, Munich, Germany.; 4 German Cancer Consortium (DKTK), Partner site Munich, A partnership between DKFZ and University Hospital LMU Munich, Munich, Germany.; 5 Section of Translational Cancer Epigenomics, Division of Translational Medical Oncology, German Cancer Research Center (DKFZ), Heidelberg, Germany.; 6 National Center for Tumor Diseases (NCT), NCT Heidelberg, A partnership between DKFZ and Heidelberg University Hospital, Heidelberg, Germany.; 7 Université de Paris Cité, Institut Necker Enfants-Malades (INEM), Institut National de la Santé et de la Recherche Médicale (Inserm) U1151, and Laboratory of Onco-Hematology, Assistance Publique-Hôpitaux de Paris, Hôpital Necker Enfants-Malades, Paris, France.; 8 Department of Internal Medicine III, University Hospital Ulm, Ulm, Germany.; 9 Heidelberg Institute for Stem Cell Technology and Experimental Medicine (HI-STEM gGmbH), Heidelberg, Germany.; 10 Division Systems Biology of Signal Transduction, German Cancer Research Center (DKFZ), Heidelberg, Germany.; 11 Division Applied Bioinformatics, German Cancer Research Center (DKFZ), Heidelberg, Germany.; 12 German Cancer Consortium (DKTK), Heidelberg, Germany.; 13 Medical Faculty and Faculty of Biosciences, Heidelberg University, Heidelberg, Germany.; 14 Hopp Children’s Cancer Center Heidelberg (KiTZ), Heidelberg, Germany.; 15 Division of Pediatric Neurooncology, German Cancer Research Center (DKFZ), Heidelberg, Germany.; 16 Biomedical Informatics, Data Mining and Data Analytics, University of Augsburg, Augsburg, Germany.; 17 Department of Medicine III, University Hospital, LMU Munich, Munich, Germany.; 18 Department of Medicine I, Medical Center – University of Freiburg, Faculty of Medicine, Freiburg, Germany.; 19 Department of Hematology and Oncology, Medical Center, Otto-von-Guericke University, Magdeburg, Germany.; 20 Hematology, Hemostasis, Oncology and Stem Cell Transplantation, Hannover Medical School (MHH), Hannover, Germany.; 21 Leibniz Institute on Aging, Fritz-Lipmann-Institute, Jena, Germany.; 22 Interventional Immunology, Leibniz Institute for Immunotherapy, Regensburg, Germany.; 23 MLL Munich Leukemia Laboratory, Munich, Germany.; 24 Clara D. Bloomfield Center for Leukemia Outcomes Research, The Ohio State University Comprehensive Cancer Center, Columbus, Ohio.; 25 Department of Hematology, Oncology, and Cancer Immunology, Charité, Universitätsmedizin Berlin, Berlin, Germany.; 26 Department of Hematology, Erasmus University Medical Center, Rotterdam, the Netherlands.; 27 Group Genome Instability in Tumors, German Cancer Research Center (DKFZ), Heidelberg, Germany.; 28 Department of Oncology, KU Leuven, Leuven, Belgium.; 29 Department of Pediatrics, Dr. von Hauner Children’s Hospital, University Hospital, LMU Munich, Munich, Germany.; 30 Faculty of Medicine, Otto-von-Guericke-University, Magdeburg, Germany.

## Abstract

Acute myeloid leukemia (AML) with complex karyotype is characterized by high genomic complexity, including frequent *TP53* mutations and chromothripsis. Genomic rearrangements can reposition active enhancers near proto-oncogenes, leading to their aberrant expression; however, a comprehensive understanding of these events in AML is still incomplete. To facilitate the discovery of such “enhancer hijacking” events, we developed Pyjacker, a computational tool, and applied it to 39 AML samples with complex karyotype. Pyjacker identified several enhancer hijacking events in AML patient samples, including aberrant expression of *MNX1*, which can result from del(7)(q22q36) and is associated with hijacking of a *CDK6* enhancer. *MNX1* activation occurred in 1.4% of patients with AML and showed significant co-occurrence with *BCOR* mutations. Through a xenograft mouse model, we demonstrated that *MNX1* is required for leukemia cell fitness. Pyjacker is an easy-to-use, accurate, and broadly applicable tool for identifying consequences of genomic events driving tumorigenesis, especially when germline genomic data are missing.

**Significance:**

This study examines the consequences of structural alterations in AML and demonstrates that proto-oncogene activation by enhancer hijacking is an understudied pathomechanism. *MNX1* overexpression demonstrates that deletions on chromosome 7q can not only lead to haploinsufficiency but also to activation of oncogenes by enhancer hijacking.

## Teaser

Systematic discovery of enhancer hijacking in complex karyotype acute myeloid leukemia via the new tool Pyjacker identifies MNX1 activation, a novel leukemogenic event that can result from 7q deletion, among other events.

## Introduction

Acute myeloid leukemia (AML) is a disease characterized by a block in differentiation and uncontrolled proliferation of myeloid progenitor cells. AML is a very heterogeneous disease and has been divided into several subgroups based on recurrent cytogenetic alterations [e.g., t(15;17)(q24.1;q21.2), inv(16)(p13.1q22), or t(8;21)(q22;q22.1)] and mutations (e.g., in *NPM1*, *TP53*, or *CEBPA*; refs. 1–3). Complex karyotype AML (ckAML) is a subtype with dismal prognosis, and there is currently an incomplete understanding of the pathogenetic mechanisms driving this disease (4). ckAML is defined by the presence of at least three cytogenetic alterations in the absence of any of the recurrent class-defining lesions. It accounts for 10% to 12% of all AML cases and is more frequent among older patients (4). ckAML samples often harbor *TP53* mutations, which are associated with a high frequency of chromothripsis, defined as the shattering of certain chromosomes and refusion in random order, resulting in highly rearranged chromosomes with loss of chromosomal material (5–7). Deletions in ckAML are more frequent than gains, and the most common deletions affect chromosome arms 5q, 7q, 17p, and 12p, whereas gains mostly occur on 8q, 11q, and 21q (4, 8, 9). According to Knudson’s two-hit hypothesis, deletions in cancer usually lead to the complete inactivation of a tumor-suppressor gene whose other copy is also inactivated, for example, by a mutation. However, apart from *TP53* on 17p, the search for tumor-suppressor genes with both copies inactivated in ckAML has been unsuccessful (4), and the current paradigm is that copy number alterations (CNA) in ckAML lead to gene dosage effects driving tumorigenesis (10), in which a higher or lower gene copy number results in a higher or lower gene expression, respectively.

Deletions of chromosomal segments on 7q are one of the most common structural alterations in AML, occurring in 10% of patients (2, 11). 7q deletions are frequently seen in ckAML but can also be found as a sole abnormality, in which it is still associated with a poor prognosis (12). The clustering of these deletions in certain regions on 7q has been used for more than 20 years as an indication for the presence of a tumor-suppressor gene within the minimally deleted region. However, the search for a gene with a second (epi)genetic hit has not been successful (13). Consequently, the most plausible explanation for these highly recurrent clustered deletions is that they lead to haploinsufficiency of genes in the deleted region, in which a lower copy number results in reduced gene expression, and that this haploinsufficiency is sufficient to drive cancer. Of note, many haploinsufficient genes located in the deleted regions of 7q encode enzymes that regulate genome-wide epigenetic patterns or transcription factors such as *CUX1*, *EZH2*, *KMT2C*, or *KMT2E* (13–15).

In addition to CNAs, structural variants (SV) can create fusion proteins or remove or create new enhancer–promoter interactions. For example, 5% of all AML cases harbor an inv(3)(q21q26.2) or a t(3;3)(q21;q26.2), which repositions the *GATA2* enhancer in close vicinity of *MECOM*, leading to aberrant *MECOM* expression and *GATA2* haploinsufficiency (16). A few other genes have been reported to be activated by enhancer hijacking in AML, including *BCL11B* in acute leukemias with a mixed phenotype (17) and *MNX1* in pediatric AML with t(7;12)(q36;p13) (18, 19). Because ckAML samples harbor many, often cytogenetically cryptic, genomic rearrangements, we hypothesized that some of them could lead to enhancer hijacking events, activating yet-undiscovered oncogenes.

Recently, several computational methods have been developed to search for genes activated by enhancer hijacking. CESAM (20), SVExpress (21), and HYENA (22) perform a linear regression of gene expression depending on the presence of breakpoints nearby. These methods have successfully identified genes recurrently activated by enhancer hijacking, but they cannot detect genes activated in only a few samples. cis-X (23) can detect enhancer hijacking events in single samples using monoallelic expression, but this method is not very flexible and requires matched normal samples, which are rarely available for AML samples. NeoLoopFinder (24) follows a very different approach: it detects neo-loops in HiC data and does not use gene expression.

In this study, we developed a new tool, “Pyjacker,” which detects putative enhancer hijacking events occurring in single samples using RNA sequencing (RNA-seq) and whole-genome sequencing (WGS) without matched normal samples. We applied Pyjacker to 39 ckAML samples using WGS and RNA-seq and identified genes known to be activated by enhancer hijacking as well as candidate genes that, to the best of our knowledge, have been previously overlooked. We focused on *MNX1*, a gene encoding a homeobox transcription factor, which is mapped to chromosome band 7q36.3, that is located outside of the most commonly deleted regions found in AML with del(7q). We profiled 31 *MNX1*-expressing cases with WGS and discovered that del(7q) can lead to hijacking of the *CDK6* enhancer driving *MNX1* expression, resulting in a shared gene expression profile with pediatric AML with *MNX1* activation. We showed that *MNX1* knockdown reduces leukemic cell fitness in patient-derived xenograft (PDX) competition assays, demonstrating its essentiality.

## Results

### Pyjacker: Detection of Enhancer Hijacking with WGS and RNA-seq

**Figure 1.**
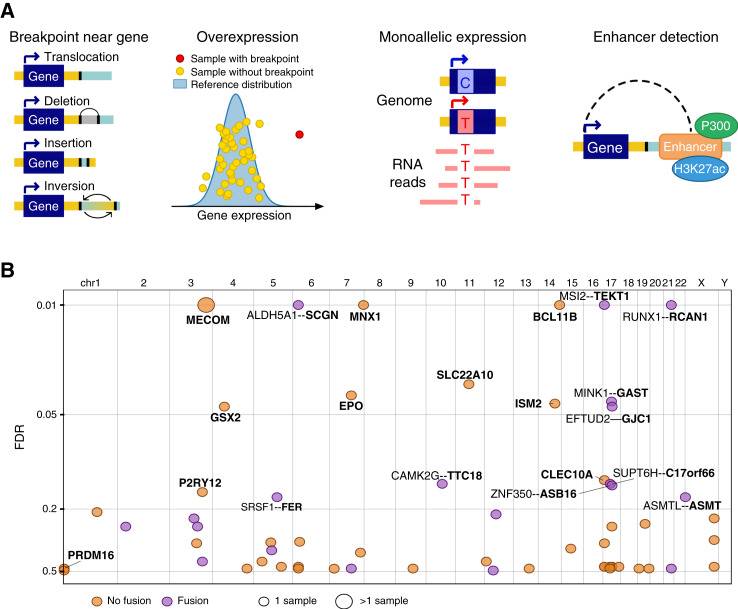
Detection of enhancer hijacking in 39 ckAML samples. **A,** Schematic representation of the main sources of information used by Pyjacker: breakpoints, overexpression, monoallelic expression, and enhancers. **B,** Scatter plot of genes identified by Pyjacker in 39 ckAML samples as being potentially activated by genomic rearrangements in one or more samples, in which the *x*-axis shows the genomic location of the genes, and the *y*-axis shows the FDR. Gene names for the enhancer hijacking candidates are written in bold, and if a fusion transcript is detected, the fusion partner is named. chr, chromosome.

We developed Pyjacker, a computational method to detect enhancer hijacking events occurring in single samples using WGS, RNA-seq, and enhancer information, without the need for matched normal samples (Supplementary Table S1). The aim of Pyjacker is to detect rearrangements that lead to a very strong overexpression of a gene that is not typically expressed or expressed only at a low level in the wild-type (WT) state. Detecting events leading to more moderate effects would not be feasible in single samples. For each gene, samples are divided into “candidate samples” which have breakpoints near the gene and “reference samples” which do not (see “Methods” section for details). Reference samples are used to compute the mean and standard deviation (SD) of the expression of this gene in the absence of enhancer hijacking, and candidate samples are tested for overexpression compared with this reference distribution (Fig. 1A). If a gene is activated by enhancer hijacking, we would expect most of the expression to come from the rearranged allele. Heterozygous SNPs are identified in the WGS data, and if these SNPs are covered in the RNA-seq data, Pyjacker tests whether the expression is mostly monoallelic (Fig. 1A). Using the breakpoint information and a list of putative enhancers, Pyjacker identifies enhancers coming close to the gene and scores the event depending on the strength of the enhancers coming close to the gene. As enhancers are cell type–specific, we used in this study chromatin immunoprecipitation sequencing (ChIP-seq) data against H3K27ac and P300 from myeloid cell lines (Supplementary Table S2) because these marks are found on active enhancers (25, 26). This enhancer information can be omitted if it is not available. The overexpression, monoallelic expression, and enhancer scores are combined into an empirical score which reflects how likely the gene is to be expressed because of a genomic rearrangement. The scores are aggregated across samples for each gene in order to give more weight to the recurrently activated genes. To estimate the FDR, “null scores” are computed by only including the “reference samples” and randomly assigning some of them to the “candidate samples,” thus reflecting the distribution of scores in the absence of enhancer hijacking. Finally, the Benjamini–Hochberg method is used to correct for multiple testing and provides a ranked list of genes putatively activated by a structural rearrangement, with corresponding FDRs. Pyjacker is flexible, and we provide an end-to-end Nextflow pipeline to run Pyjacker, starting from BAM files. We note that fusion transcripts can also result in monoallelic overexpression, when the 3′ fusion partner is not normally expressed, although this would be a different mechanism than enhancer hijacking. Various methods can be used to detect fusion transcripts from RNA-seq data, like STAR-Fusion (27) or Arriba (28). If a list of fusions generated by these methods is given as input to Pyjacker, it will annotate candidate genes with the fusion status, allowing the identification of true enhancer hijacking events. Because Pyjacker needs reference samples without breakpoints near a gene to estimate the reference expression distribution, it should be run with at least 10 samples as input but works best with large cohorts. We tested Pyjacker on two existing datasets with known enhancer hijacking events: 10 AML cell lines and 120 medulloblastoma samples (29, 30). Pyjacker identified known events, like the activation of *MECOM* (16), *MNX1* (31), and *MN1* (32) in some AML cell lines and of *GFI1*, *GFI1B*, and *PRDM6* in some medulloblastoma samples, as previously reported by Northcott and colleagues (refs. 29, 30; Supplementary Tables S3–S5). cis-X also identified GFI1, GFI1B, and PRDM6, but these events did not particularly stand out among the many candidate genes reported, whereas they were all among the top 10 genes identified by Pyjacker due to Pyjacker’s aggregation of scores across samples, which gives more weight to recurrently activated genes.

### Putative Enhancer Hijacking Events in 39 ckAML Samples

We profiled 39 ckAML samples with WGS and RNA-seq. These were diagnostic blood or bone marrow (BM) samples from patients enrolled in the ASTRAL-1 clinical trial which included older patients with AML (median age: 77 years, Supplementary Table S6; ref. 33). These samples carried some of the alterations most frequently found in ckAML (34), including biallelic *TP53* alterations (64%, *N* = 25), del(7q) (69%, *N* = 27), del(5q) (67%, *N* = 26), and chromothripsis (43%, *N* = 17; Supplementary Fig. S1; Supplementary Tables S6–S10).

Pyjacker was applied to these 39 samples and detected 19 candidate genes with an FDR < 20% (Fig. 1B; Supplementary Table S11). Among them were many of the genes which had previously been reported to be activated by enhancer hijacking in AML, including *MECOM* (two samples), *MNX1* (one sample), and *BCL11B* (one sample). In addition, Pyjacker identified several genes that had not been reported before and which represent interesting candidate oncogenes to be verified in future studies. For 9 of the 19 genes, no fusion transcript was detected, suggesting enhancer hijacking as the underlying activation mechanism: *MECOM*, *MNX1*, *BCL11B*, *SLC22A10*, *EPO*, *ISM2*, *GSX2*, *CLEC10A* and *P2RY12*. In order to evaluate how recurrent the upregulation of these genes is in AML, we used datasets from The Cancer Genome Atlas (TCGA)-LAML (1), BEAT-AML (35), and TARGET-AML (36) cohorts. We found that most of the genes identified by Pyjacker were recurrently overexpressed in these other AML cohorts, albeit at low frequencies (Supplementary Fig. S2). However, some genes were not found overexpressed in these three other AML cohorts, which suggests either that their activation is a very rare event in AML, that they are false positives, or that their overexpression in our cohort was a passenger event of chromothriptic rearrangements. For example, the activations of *TEKT1* (in 16PB3075) and of *SLC22A10* (in 15KM20146) were due to complex rearrangements which also contained SVs within *TP53* (Supplementary Fig. S3A–S3F). Thus, these rearrangements might have been selected for because of the *TP53* disruption rather than *TEKT1* or *SLC22A10* activation.

### Activation of *MECOM* and Its Homolog *PRDM16* by the *GATA2* Enhancer

**Figure 2.**
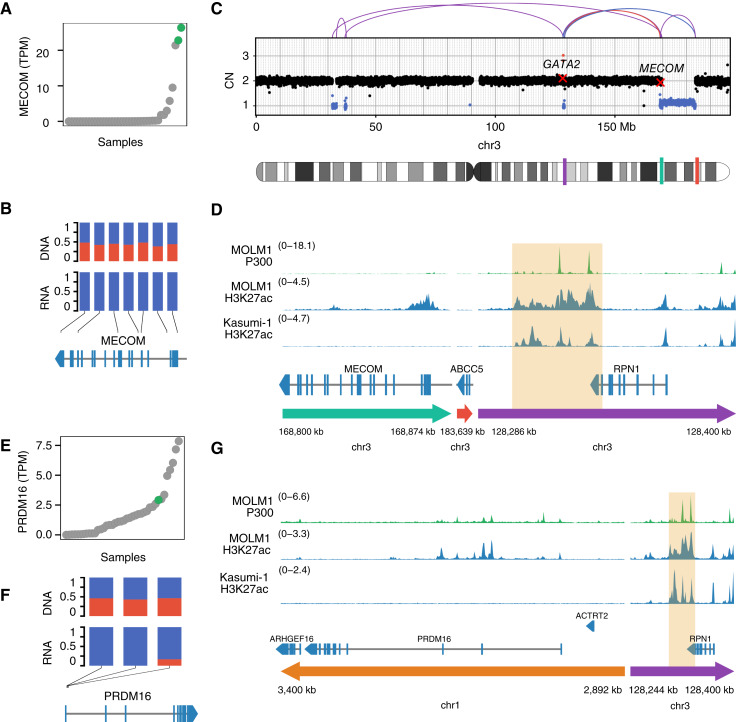
Activation of *MECOM* and its homolog *PRDM16* by a *GATA2* enhancer. **A,** Expression of *MECOM* in all samples in TPM, ranked by expression of *MECOM*, in which samples 15PB19457 and 15KM20146 with breakpoints near *MECOM* are highlighted in green. **B,** Variant allele frequencies in WGS (DNA) and RNA-seq for SNPs in *MECOM* for sample 15PB19457 (major allele frequencies in blue and minor allele frequencies in red). **C,** Copy numbers (CN) and SVs on chromosome 3 for sample 15PB19457. Copy number losses are indicated in blue, and gains in red. SVs are shown as arcs at the top, in which the color indicates the orientation of the breakpoint: blue for deletion, red for duplication, and purple for inversion. In the chromosome ideogram, the three regions that are displayed with a zoom-in in (**D**) are highlighted in colors, with colors matching the arrows in (**D**). **D,** ChIP-seq tracks for P300 and H3K27ac in the myeloid cell lines MOLM-1 and Kasumi-1 in the region around *MECOM* for the rearranged chromosome of sample 15PB19457. The putative enhancer is highlighted in orange. **E,** Expression of *PRDM16* in all samples, ranked by *PRDM16* expression, in which sample 16KM11270 with a breakpoint near *PRDM16* is highlighted in green. **F,** Variant allele frequencies in WGS (DNA) and RNA-seq for SNPs in *PRDM16* in TPM for sample 16KM11270 (major allele frequency in blue and minor allele frequency in red). **G,** ChIP-seq tracks for P300 and H3K27ac in the myeloid cell lines MOLM-1 and Kasumi-1 in the region around PRDM16 on the rearranged chromosome of sample 16KM11270. The putative enhancer is highlighted in orange. chr, chromosome.

The only gene identified by Pyjacker in more than one sample from this cohort was *MECOM*, found to be monoallelically overexpressed in two samples (Fig. 2A and B; Supplementary Fig. S4A–S4C). In both cases, the rearrangements were more complex than those found in samples with inv(3) or t(3;3) AML, which are the most frequent rearrangements responsible for *MECOM* activation. One sample had chromothripsis on chromosome 3 (Fig. 2C), whereas the other one had several rearrangements between chromosome 3 and chromosome 14 (Supplementary Fig. S4A). Even though these rearrangements were very complex, they still resulted in the juxtaposition of *MECOM* to a *GATA2* enhancer (next to *RPN1*) harboring enhancer marks in myeloid cell lines (Fig. 2D), which is the same enhancer that activates *MECOM* in the more common inv(3) and t(3;3) (16). Interestingly, the *GATA2* enhancer was also reported by Pyjacker to activate *PRDM16* in another sample (16KM11270) through a translocation t(1;3)(p36;q21) (Fig. 2E–G). *PRDM16* is a homolog of *MECOM* (also known as *PRDM3*; ref. 37), and they are both H3K9me1 methyltransferases (38), so their overexpression could play a similar role in AML. This t(1;3) translocation has been reported before as a rare event (37), and *PRDM16* has also recently been reported to be overexpressed as a result of a rare t(1;2)(p36;p21) translocation (39). Even though the expression of *PRDM16* was monoallelic in this sample (Fig. 2F), which is a strong indicator of activation by enhancer hijacking, the FDR reported by Pyjacker was high (47%) because several samples without breakpoints near *PRDM16* had a higher expression than this sample (Fig. 2E). *MECOM* is also expressed in samples without breakpoints nearby (40), although to a lesser extent, suggesting an additional activation mechanism for *MECOM* and *PRDM16* besides enhancer hijacking.

### Aberrant *EPO* Expression and *EPO* Receptor Amplification in Acute Erythroleukemia

**Figure 3.**
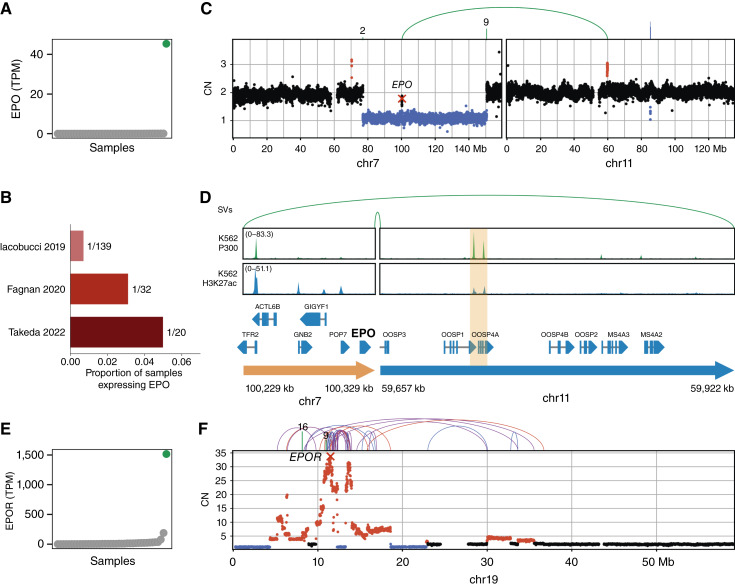
Aberrant *EPO* expression might cooperate with *EPOR* amplification in AEL. **A,***EPO* expression in all samples in TPM, with sample 15KM18875 with *EPO* overexpression highlighted in green. **B,** Proportion of samples with nonzero *EPO* expression in three AEL cohorts profiled with RNA-seq (47–49). **C,** Copy numbers (CN) and SVs on chromosome 7 (containing *EPO*) and chromosome 11 in sample 15KM18875. Copy number losses are indicated in blue, and gains in red. SVs are shown at the top, with arcs connecting breakpoints or lines indicating the chromosome of the other side of the breakpoint (for **C**, **D**, and **F** the colors of SVs indicate the orientation: blue for deletion, red for duplication, purple for inversion, and green for interchromosomal SV). **D,** A 300 kb circular piece of DNA containing *EPO* and a putative enhancer (highlighted in orange), with P300 and H3K27ac peaks in the erythroid cell line K562. **E,***EPOR* expression in TPM in all samples, with sample 15KM18875 highlighted in green. **F,** Copy numbers and SVs on chromosome 19 for sample 15KM18875. chr, chromosome.

Among the genes identified by Pyjacker, an interesting candidate was *EPO*. To our knowledge, this gene has never been reported to be activated by enhancer hijacking in human leukemias, although it has been found to be overexpressed due to genomic rearrangements in a mouse model of erythroleukemia (41, 42). *EPO* is not expressed in normal hematopoietic cells, but it is instead produced in the kidneys when blood oxygen levels are low, and it stimulates red blood cell proliferation by binding to its receptor [*EPO* Receptor (*EPOR*)] and activating the JAK/STAT pathway (43–45). Because *EPO* promotes survival, proliferation, and differentiation of erythroid progenitor cells (46), it may drive acute erythroleukemia (AEL), a rare subtype of AML enriched for complex karyotypes. In this ckAML cohort, the AEL sample 15KM18875 had high *EPO* expression (Fig. 3A). Although no samples from the TCGA-LAML, BEAT-AML, and TARGET-AML cohorts expressed *EPO*, we found that among three AEL cohorts profiled with RNA-seq (47–49), one sample from each cohort expressed *EPO* (Fig. 3B), indicating that *EPO* expression is a rare but recurrent event in AEL. In sample 15KM18875, a 100 kb region on chromosome 7 around *EPO* was duplicated and fused with a region on chromosome 11 (Fig. 3C) such that an extrachromosomal circular DNA (eccDNA) was formed (Fig. 3D). eccDNAs are rather common in cancer, but they are often amplified, whereas sample 15KM18875 displayed an average copy number of less than one eccDNA per cell. This eccDNA is therefore subclonal, but it is unclear whether most cells have one copy or whether a small percentage of cells contain numerous copies. The chromosome 11 portion of the eccDNA contains a putative enhancer with P300 and H3K27ac peaks in the leukemic cell line K562 with erythroid features (50), so this enhancer might be responsible for the activation of *EPO* in this sample. In addition to high *EPO* expression, we also observed very high expression of the *EPOR* in 15KM18875 (Fig. 3E), which was due to a massive amplification of *EPOR* on chromosome 19 (Fig. 3F). Chromosome 19 harbored patterns of chromothripsis, as well as foldback inversions, suggesting that the amplifications were caused by breakage–fusion–bridge cycles (51). Rearrangements of *EPOR* are well-known in acute lymphoblastic leukemia (52), and amplification of *EPOR* has recently been reported as a recurrent driver event in AEL (49). High *EPOR* expression could make the cells very sensitive to *EPO*, thus increasing the fitness advantage provided by endogenous *EPO* expression by the leukemic cells. In both the Iacobucci and colleagues (47) and Fagnan and colleagues (48) cohorts, the sample with *EPO* expression also had outlier high *EPOR* expression, indicating that *EPO* is recurrently overexpressed together with *EPOR*.

### The Homeobox Genes *GSX2* and *MNX1* Can Be Activated by Atypical Rearrangements

**Figure 4.**
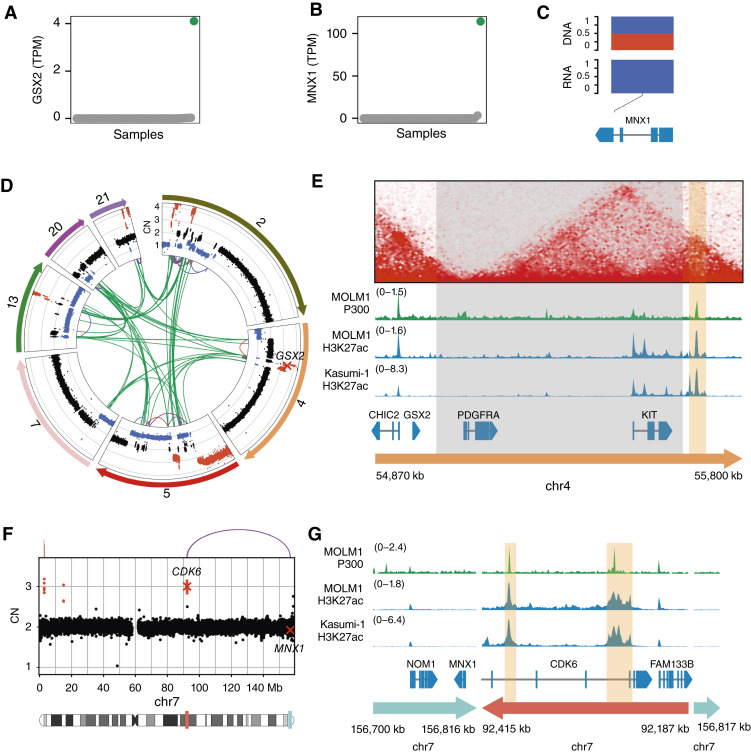
The homeobox genes *GSX2* and *MNX1* can be activated by atypical mechanisms. **A,***GSX2* expression in all samples in TPM, with sample 16PB5693 with *GSX2* expression highlighted in green. **B,***MNX1* expression in all samples in TPM, with sample 15PB8708 with *MNX1* overexpression highlighted in green. **C,** Variant allele frequencies in WGS and RNA-seq for an SNP in *MNX1* in sample 15PB8708 (major allele frequency in blue and minor allele frequency in red). **D,** Circos plot showing CNAs and SVs in sample 16PB5693 for the chromosomes involved in a chromothripsis event. Copy number (CN) losses are indicated in blue, and gains in red. SVs are shown as arcs at the center, with interchromosomal breakpoints in green, duplications in red, deletions in blue, and inversion in purple. **E,** HiC data from HSPCs (19) and ChIP-seq data from myeloid cell lines in the region around *GSX2*. The putative enhancer is highlighted in orange, and the region in gray is deleted in sample 16PB5693. **F,** CNs and breakpoints on chromosome 7 for sample 15PB8708. In the chromosome ideogram, regions highlighted in red and teal correspond to the regions shown in (**G**), with matching colors. **G,** ChIP-seq tracks for P300 and H3K27ac in the myeloid cell lines MOLM-1 and Kasumi-1 in the region around *MNX1* on the rearranged chromosome of sample 15PB8708. Enhancers of the CDK6 region are highlighted in orange. chr, chromosome.

Among the top Pyjacker hits were two homeobox genes, *GSX2* and *MNX1*, which were overexpressed in samples 16PB5693 and 15PB8708, respectively. Both samples have breakpoints near the respective genes, and in sample 15PB8708, heterozygous SNPs in *MNX1* confirmed monoallelic expression (Fig. 4A–C). Homeobox genes are often upregulated in AML (53), so the activation of homeobox genes by enhancer hijacking could be a driver event. Both *GSX2* and *MNX1* are known to be activated by rare but recurrent translocations to the *ETV6* locus: *GSX2* by t(4;12)(q11-q12;p13) in adult AML (54) and *MNX1* by t(7;12)(q36;p13) in pediatric AML (19). In this study, however, *GSX2* and *MNX1* were activated by atypical mechanisms. Sample 16PB5693 was affected by a chromothripsis event involving multiple chromosomes, and several genomic segments, including *GSX2*, were amplified (Fig. 4D). In the WT state, the putative enhancer is located less than 1 Mb away from *GSX2* but in a different topologically associating domain (TAD; Fig. 4E). In sample 16PB5693, a deletion removed the TAD boundary, which likely enabled *GSX2* to interact with the enhancer. In addition to *GSX2* upregulation, the recurrent t(4;12) translocation frequently leads to *PDGFRA* activation and to an *ETV6::CHIC2* fusion transcript (55). Sample 16PB5693 only had *GSX2* expression without *PDGFRA* expression and without fusion transcript, suggesting that *GSX2* expression is the driving event. In sample 15PB8708, a 230 kb segment in the *CDK6* region, containing two putative enhancers, was duplicated and inserted next to *MNX1* (Fig. 4F and G). The breakpoints were verified by genomic PCR (Supplementary Fig. S5A–S5C; Supplementary Table S12). This hematopoietic super-enhancer has already been reported to be involved in enhancer hijacking events in AML, activating *BCL11B* (17) or *EVI1* (56). *MNX1* was expressed in a rather high proportion of the TCGA-LAML and BEAT-AML cohorts (2/179 and 17/707 samples with *MNX1* expression, respectively), and in some cases, the karyotype contained rearrangements near *MNX1* on 7q36 [del(7)(q21q36) for TCGA-AB-2847, del(7)(q22q36) for BA2921, and t(7;7)(q22;q36) for BA2802], indicating that *MNX1* expression could be due to enhancer hijacking in some of these samples.

### *MNX1* Is Expressed in 1.4% of all AML Cases, Often with del(7)(q22q36)

**Figure 5.**
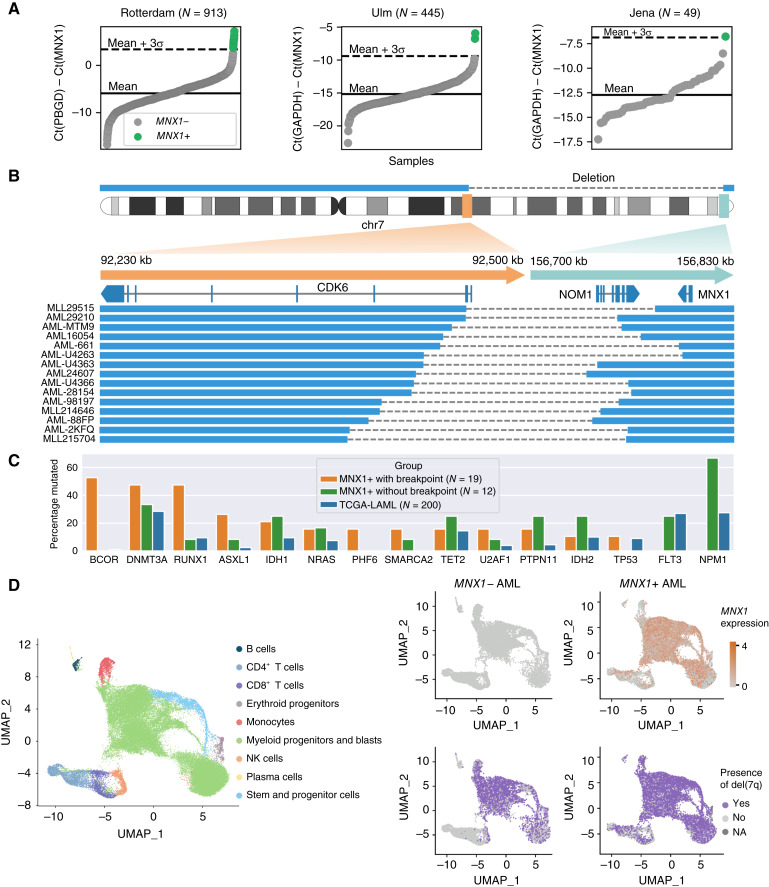
*MNX1* is expressed in 1.4% of all AML cases, often with del(7)(q22q36). **A,** qRT-PCR screen for *MNX1* expression in three AML cohorts (Rotterdam, Ulm, and Jena). **B,** Fifteen *MNX1*-expressing samples with del(7)(q22q36) profiled with WGS, with a zoom-in around the breakpoints (hg19 reference). The blue rectangles indicate the genomic regions that are retained, and dashed lines represent breaks. **C,** Percentage of samples with mutations in frequently mutated genes for *MNX1*+ samples with breakpoints near *MNX1*, *MNX1*+ samples without breakpoints, and TCGA-LAML samples. **D,** scRNA-seq analysis for *MNX1*+ and control del(7q) AML samples. Left, UMAP showing cell type labels of 53,479 cells integrated across eight patients. Right, UMAP highlighting *MNX1* expression (top) and the presence of a del(7q) (bottom) as predicted for patients with del(7q) (*n* = 4) and patients with del(7q) and *MNX1* activation (*n* = 4). chr, chromosome; UMAP, Uniform Manifold Approximation and Projection.

To estimate the frequency of aberrant *MNX1* expression in AML cases, we performed an unbiased qRT-PCR screen of three different AML cohorts (Rotterdam, Ulm, and Jena; Fig. 5A). In a total of 2,293 cases across five cohorts [three qRT-PCR cohorts and public RNA-seq data from TCGA-LAML (1) and BEAT-AML (35)], we estimated the frequency of *MNX1*-expressing samples to be 1.4% of all AML cases (Supplementary Table S13). We also screened del(7q) and ckAML cases and found a higher proportion of *MNX1*-expressing samples in these selected groups [8.70% in del(7q) and 2% in ckAML; Supplementary Table S13].

We performed WGS on 23 *MNX1*-expressing primary AML samples (whole blood or BM) taken at diagnosis, which we combined with WGS data of eight samples provided by the Munich Leukemia Laboratory (MLL), resulting in a total of 31 *MNX1*-expressing samples profiled with WGS. The data for the eight samples from the MLL were processed with the MLL pipeline as previously described (57), whereas the 23 other samples were processed in the same way as the 39 ckAML samples described in this article. Fifteen samples had a large del(7)(q22q36) starting within *CDK6* and ending before *MNX1* (Fig. 5B; Supplementary Table S14), indicating that *MNX1* could be activated by an enhancer in the *CDK6* region in those samples. Interestingly, this is the same region that is duplicated and inserted next to *MNX1* in sample 15PB8708 (Fig. 4F and G). Four samples had other rearrangements near *MNX1*, including a smaller del(7q) between the T-cell receptor β locus and *MNX1* (Supplementary Figs. S6A–S6D, S7A, and S7B), which supports the notion that other enhancers apart from *CDK6* might activate *MNX1*. Indeed, we had previously found a *MYB* enhancer in GDM-1 cells (31) and an *ETV6* enhancer in t(7;12)(q36;p13) pediatric AML (19) to drive aberrant *MNX1* expression. Twelve samples had no rearrangements near *MNX1*, suggesting that *MNX1* may also be activated through other mechanisms.

Samples with *MNX1* rearrangements had a unique mutational spectrum with an absence of *NPM1* and *FLT3* mutations (0/19), as well as a very high frequency of *BCOR* mutations (10/19), which are usually rare in AML (2/200 in TCGA-LAML), although they have recently been reported to have a frequency of about 10% in AML with del(7q) (Fig. 5C; Supplementary Table S15; ref. 11). *BCOR* mutations were accompanied by *BCORL1* (2/10) and *NCOR2* (1/10) mutations indicating a potential synergistic effect of multiple hits on this gene family. We also found *NCOR1* (1/9) and *NCOR2* (1/9) mutations in *BCOR* WT cases, indicating that they might play a similar role as *BCOR* mutations. *MNX1*-expressing samples without breakpoints near *MNX1* did not share this mutational landscape but had a particularly high frequency of mutations in *NPM1* (8/12; ref. 58). *MNX1*, however, has not been shown to be in the *NPM1* gene signature in previous studies. In pediatric AML, *MNX1* can be expressed as a result of a translocation t(7;12), which very often co-occurs with trisomy 19 (19). However, trisomy 19 was not found in this cohort of adult *MNX1*-expressing samples.

We profiled 22/31 *MNX1*-positive (*MNX1*+) samples with RNA-seq and found that they had a different gene expression signature, depending on whether the sample had a breakpoint near *MNX1* or not (Supplementary Figs. S8A–S8F and S9; Supplementary Table S16). *MNX1*-rearranged samples had a gene expression signature similar to t(7;12)(q36;p13) pediatric AML (19, 59, 60), with, for example, an upregulation of *AGR2*, *KRT72*, and *KRT73.* Downregulated genes included several key cancer- and hematopoiesis-associated genes: *HLX*, *TFEC*, *GFI1*, *GAPT*, *SPRY2*, *TLE4*, *ACVR1B*, *BIK*, *EVI2B*, *PIK3CG*, *INPPL1* (*SHIP2*), *MYD88*, *MACC1*, *CSF3*, and *CD177*. *MNX1*–non-rearranged samples had a different gene expression signature with a significant upregulation of *HOXA13*, *CCL1*, and *CX3CR1* and a downregulation of *DLK1* and *DDIT4L*. *MNX1* expression was slightly lower than in *MNX1*-rearranged cases, and some of the downregulated genes also showed intermediate levels in *MNX1*–non-rearranged samples.

Next we performed single-cell RNA-seq (scRNA-seq) on eight AML samples [four *MNX1*+ and four *MNX1*-negative (*MNX1*−) with del(7q); Supplementary Fig. S10] to investigate the expression of *MNX1* and the presence of del(7q) at the single-cell level. We integrated scRNA-seq data for 53,479 cells across all patients and annotated the cell types by projecting the data onto a reference atlas (Fig. 5D; ref. 61). We mainly captured myeloid progenitors and leukemic blasts, consistent with the disease phenotype. We observed that del(7q) was present in virtually all leukemic blasts across both groups (*MNX1*− and *MNX1*+), suggesting that this genomic alteration was an early event in leukemogenesis in these patients. In *MNX1*+ cases, *MNX1* was constitutively expressed in all blasts, indicating that cells with *MNX1* activation might have a proliferative advantage.

### Putative Enhancers in the *CDK6* Region Interact with *MNX1* in del(7q) AML

**Figure 6.**
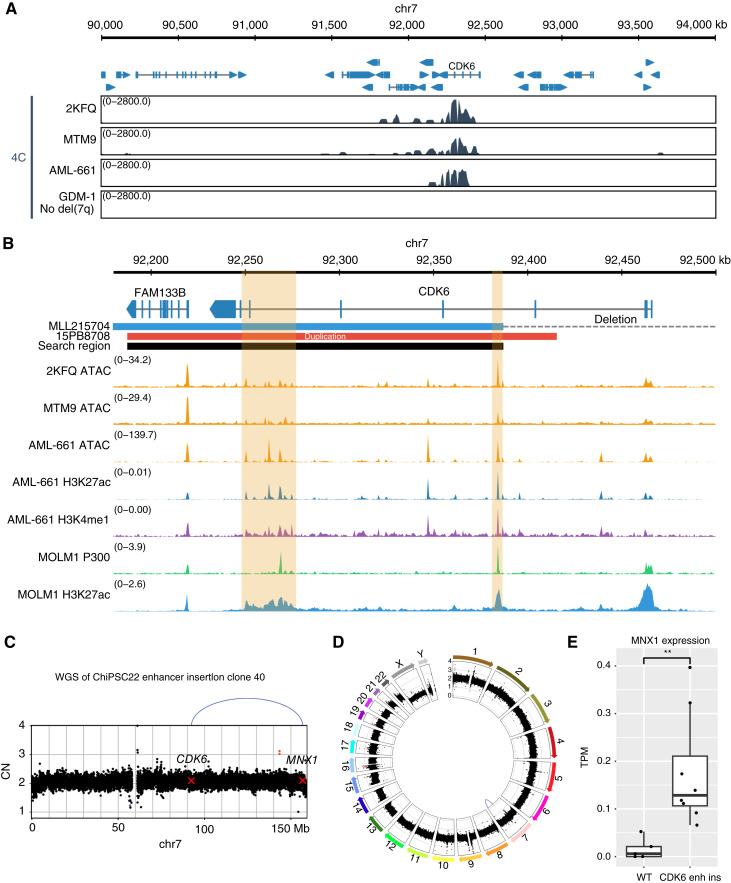
Putative enhancers in the *CDK6* region interact with *MNX1* in del(7q) AML. **A,** Chromatin interaction detected with 4C in the region around *CDK6* using *MNX1* as viewpoint, for three different del(7)(q22q36) samples and one control sample (GDM-1 cell line) without del(7q). **B,** The 200 kb search region based on the enhancer duplication (sample 15PB8708) and the sample with the leftmost deletion (MLL215704), with tracks for enhancer marks: ATAC-seq in del(7q) samples MTM9 and 2KFQ, ATAC-seq and ACT-seq against H3K27ac and H3K4me1 in the PDX sample AML-661 derived from a del(7q) patient, and ChIP-seq against P300 and H3K27ac in the MOLM-1 cell line. The putative enhancers are highlighted in orange. **C,** Copy number (CN) profile and SVs on chromosome 7 in the engineered cell line validating the insertion of the 1 kb region. **D,** Circos plot for the same cell line showing the absence of other rearrangements. Copy number losses are indicated in blue, and gains in red. SVs are shown as arcs at the center, with interchromosomal breakpoints in green, duplications in red, deletions in blue, and inversion in purple. **E,***MNX1* expression in TPM for the parental ChiPSC22 HSPCs (*n* = 5, from independent differentiation experiments) compared with the engineered cell with the enhancer insertion (enh ins; *n* = 8, from independent differentiation experiments for two different cell lines). **, *P* < 0.01 using a two-sided *t* test. chr, chromosome.

Because most samples with *MNX1* activation have breakpoints in *CDK6*, we set out to identify the corresponding enhancer. To investigate whether *MNX1* may interact with the *CDK6* locus in selected del(7)(q22q36) samples, we performed circular chromosome conformation capture (4C) using a 5′ part of *MNX1* as viewpoint in two primary AML samples (2KFQ and MTM9) and one human PDX (AML-661) with del(7q). In all three cases analyzed, we detected interactions between *MNX1* and the *CDK6* locus (Fig. 6A). We confirmed these interactions by reciprocal 4C using the *CDK6* locus as viewpoint (Supplementary Fig. S11). We further narrowed down the *CDK6*-derived enhancer to roughly 200 kb by combining the genomic information from the *CDK6* duplication of ckAML sample 15PB8708 and from the deletion margins of the del(7q) samples (Fig. 6B). Open chromatin profiling by Assay for Transposase-Accessible Chromatin by Sequencing (ATAC-seq) and enhancer mark profiling by antibody-guided chromatin tagmentation sequencing (ACT-seq) in two patient samples and one PDX sample with del(7)(q22q36) revealed several enhancer candidates, two of which coincided with P300 and H3K27ac peaks in the MOLM-1 cell line (Fig. 6B). Comparing intensities of common peaks, we considered the rightmost enhancer (chr7:92384001–92385000, hg19) located immediately at the deletion border as the strongest candidate and inserted it as a homology-directed repair donor template via CRISPR/Cas close to *MNX1* into one of the two chromosomes 7 of the induced pluripotent stem cell (iPSC) line ChiPSC22 (Fig. 6C and D). Two heterozygous cell lines were confirmed by WGS. Upon differentiation into hematopoietic stem and progenitor cells (HSPC), the engineered, but not the WT, HSPCs showed *MNX1* expression as validated by RNA-seq, although at a significantly lower level than in patient samples (Fig. 6E). Therefore, this rightmost enhancer is not sufficient to induce the high *MNX1* expression observed in del(7)(q22q36) patients alone and might require additional enhancers from this region. To recapitulate the genomic configuration of *MNX1* expressors with del(7q), we generated a heterozygous del(7)(q22q36) in the iPSC/HSPC model. However, del(7q) iPSCs could not be differentiated into HSPCs and therefore did not show *MNX1* activation. Taken together, *MNX1* activation in del(7q)(q22q36) AML could be traced to a region of 200 kb, including parts of *CDK6*. Identifying the precise location of the enhancer(s) will require future work.

### Knockdown of *MNX1* Reduces Tumor Load of AML PDX Cells *In Vivo*

**Figure 7.**
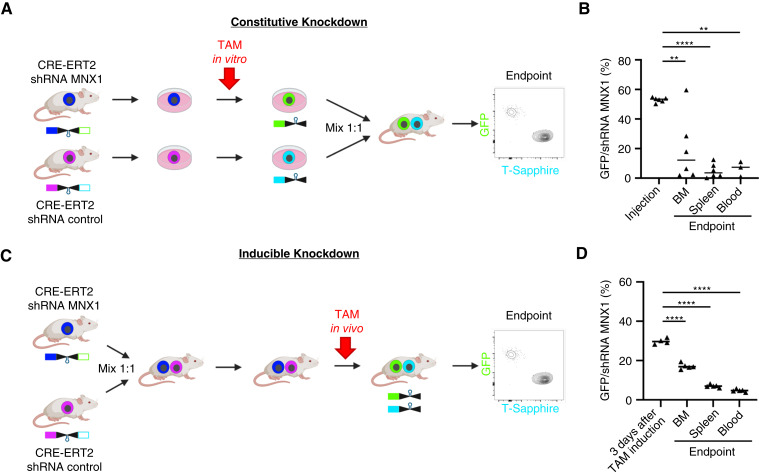
Knockdown of *MNX1* reduces tumor load of AML PDX cells *in vivo*. **A,** Scheme depicting the experimental setup of the *in vivo* constitutive experiment. AML-661 PDX cells expressing the cassettes for both CRE-ERT2 and the shRNA addressing *MNX1* or a control gene were amplified in mice. Fresh PDX cells were stimulated with TAM (single dose, 200 nmol/L, 72 hours) to induce the knockdown *in vitro*. Cells with knockdown were enriched by flow cytometry gating on the respective fluorochrome markers GFP (knockdown of *MNX1*) and T-Sapphire (control knockdown). The two populations were mixed at a 1:1 ratio and injected into mice. The ratio between both populations was measured at advanced leukemic disease in different organs (more than 60% hCD33^+^ cells in PB). **B,** The results of the experiment described in (**A**) using five mice. **C,** Scheme depicting the experimental setup of the *in vivo* inducible experiment. The cell populations described in (**A**) were mixed in a 1:1 ratio and injected into 13 mice. Fourteen days after injection, three mice were sacrificed (*N* = 3) to quality control the 1:1 ratio of the two cell populations by flow cytometry. TAM (50 mg/kg) was orally administered to the 10 remaining mice. Five mice were sacrificed 3 days later to measure the rate of shRNA induction by TAM. At an advanced stage of leukemia, the remaining five mice were sacrificed to determine the ratio between the control vs. *MNX1* knockdown populations. **D,** The results of the experiment described in (**C**). *P* values determined by a one-tailed unpaired *t* test. **, *P* < 0.01; ****, *P* < 0.0001.

After having demonstrated that *MNX1* can be activated by enhancer hijacking in AML, we investigated whether *MNX1* plays a role in the maintenance of established leukemias. To approximate the clinical situation, we studied a patient’s AML cells growing in mice using PDX model AML-661 which harbors a del(7)(q21.13;q36.3) and expresses *MNX1*. Using lentiviruses, we stably expressed two different constructs in each cell, namely CRE-ERT2 in which CRE becomes activated by addition of tamoxifen (TAM) and a CRE-inducible short hairpin RNA (shRNA) cassette in two different versions, for knockdown of either *MNX1* or a control gene. The two knockdown constructs were molecularly marked by different fluorochromes to distinguish the two populations by flow cytometry, before and after induction of the knockdown by TAM. *In vivo* experiments were performed in a competitive approach, injecting a mixture of cells with *MNX1* or control knockdown in a 1:1 ratio into the same mouse (Fig. 7A; ref. 62). In the first, constitutive experiment, *MNX1* and control knockdowns were induced by TAM *in vitro* before transplantation of PDX cells into mice (Fig. 7A). After a period of several weeks of leukemic growth in mice, allowing initial engraftment in the orthotopic niche and later following substantial proliferation within the BM and dissemination to extramedullary sites in the blood, cells with *MNX1* knockdown showed a pronounced disadvantage compared with cells with control knockdown in all organs studied (Fig. 7B), suggesting that lack of *MNX1* reduced fitness of PDX AML-661 cells *in vivo*. To distinguish the effect of *MNX1* knockdown on engraftment versus proliferation, a second experiment was performed in which *MNX1* and control knockdowns were induced after the leukemic disease was readily established in mice by systemic treatment of mice with TAM (Fig. 7C). Again, cells with *MNX1* knockdown had a remarkable disadvantage over control cells, most prominently in spleen and peripheral blood (PB), indicating that *MNX1* knockdown reduced *in vivo* growth of AML-661 cells (Fig. 7D). As the effect was stronger in the first constitutive compared with the second inducible experiment, both biologic processes of cell engraftment and *in vivo* proliferation might rely on expression of *MNX1*.

## Discussion

Reports have indicated enhancer hijacking as a mode of proto-oncogene activation in AML (16, 17, 19). In this study, we developed Pyjacker, a computational method for the systematic detection of enhancer hijacking events using WGS, RNA-seq data, and enhancer information. Pyjacker is versatile and applicable to many cancer types, but in this study we focused on ckAML. In 39 ckAML samples, Pyjacker detected 19 genes putatively activated by SVs in at least one sample with an FDR <20%. This indicates the importance of enhancer hijacking in ckAML, although it is not as frequent as the most recurrent deletions in 5q and 7q. We found known genes activated by enhancer hijacking such as *MECOM*, *BCL11B*, and *MNX1* and identified multiple potential novel oncogenes in AML. This is in line with a recent study using HiC and WGS data for the detection of neo-loops in 25 AML samples, which also identified enhancer-promoter, as well as silencer-promoter interactions in AML, together suggesting an overlooked repertoire of leukemic events (63). That study used NeoLoopFinder to identify hundreds of new 3D contacts across SVs, much more than the number of putative enhancer hijacking events that we identified with Pyjacker in this study. We believe that focusing on strong gene overexpression is more robust to identify critical oncogenes than relying on HiC data, which might include many false positives where the gene expression does not vary a lot. In addition, RNA-seq data is more commonly available than HiC data, especially for large cohorts, which makes Pyjacker more widely applicable.

*GSX2* is a homeobox gene which is overexpressed in AML samples with the rare t(4;12)(q12;p13) translocation (54), but this translocation also often leads to overexpression of *PDGFRA* and fusions involving *ETV6*, the most frequent being *ETV6::CHIC2* (55). In this study, we found a different rearrangement causing only *GSX2* overexpression without these additional effects, suggesting that activation of *GSX2* might be the driver event in the t(4;12) translocation and that understanding the role of *GSX2* in leukemogenesis could be important for therapeutic targeting.

*EPO* is another putative novel oncogene activated by enhancer hijacking in a small fraction of AEL samples. *EPO* had already been found to be activated by structural rearrangements in a mouse model of erythroleukemia, resulting in growth factor independence (41, 42). In this study, we found one human AEL sample with *EPO* overexpression linked to a genomic rearrangement. Although *EPO* activation is rare, it seems to be recurrent in AEL, as we identified it in three additional cohorts (47–49), including a previously reported out-of-frame fusion transcript *YWHAE*::*EPO* which was probably selected because it led to *EPO* upregulation (48). In addition, *EPO* overexpression seems to cooperate with amplifications of the gene coding for its receptor, a phenomenon recently described in AEL (49), because expression of *EPO* was found to co-occur with *EPOR* amplification.

Some identified genes were not found to be expressed in other cohorts, indicating that they may be very rare driver events, false positives, or passenger events which were selected for as part of a complex rearrangement. For example, both *TEKT1* and *SLC22A10* overexpression co-occurred with complex genomic rearrangements involving multiple chromosomes, which also disrupted *TP53*.

We focused validation experiments on *MNX1* because it was, among the top Pyjacker hits, the second (following *MECOM*) most recurrently expressed gene in other cohorts (1, 35). We found that *MNX1* is expressed in 1.4% of all AML cases, often with del(7)(q22q36). Activation of *MNX1* with del(7q) had been reported before (64), and in his study we showed that the mechanism underlying the activation is a hijacking of a *CDK6* enhancer. Del(7q) is a recurrent event in AML and currently explained by haploinsufficiency of one or several genes, including *EZH2*, *KMT2C*, *KMT2E*, and *CUX1* (11, 13–15). Our findings show that, in addition to haploinsufficiency of the deleted genes, del(7q) can also lead to enhancer hijacking of *MNX1*. In one sample, a *CDK6* enhancer was duplicated and inserted next to *MNX1*, without deletion, which makes it very likely that *MNX1* activation is important for leukemogenesis and not merely a passenger side effect of del(7q). *MNX1* upregulation had previously been observed in infant AML with t(7;12)(q36;p13) and was shown to transform fetal HSPCs in mice (19, 65). In this study, we showed that both constitutive and *in vivo* inducible knockdown of *MNX1* in competitive assays in an AML PDX model greatly reduced the fitness of the leukemic cells, which demonstrates that *MNX1* is a dependency gene in adult AML. However, only 8% of del(7q) AML cases have *MNX1* expression, so enhancer hijacking cannot explain all del(7q) cases, and haploinsufficiency of genes in the deleted region remains the likely main consequence of del(7q). We found that this subgroup of *MNX1*-rearranged adult AML samples have a unique mutational profile with a much higher rate of *BCOR* mutations (53%) than other AML samples (1%) as well as del(7q) AML (10%; ref. 11). This differs from pediatric AML cases with t(7;12) which do not have these co-occurring *BCOR* mutations but instead frequently harbor trisomy 19 (19), an alteration that we did not detect in adult *MNX1*-rearranged cases. This group of adult *MNX1*-rearranged patients had a gene expression signature that is similar to t(7;12) pediatric AML (59), suggesting that therapeutic strategies targeting *MNX1* could be jointly investigated for both pediatric and adult *MNX1*-rearranged AML cases. Suppression of key genes involved in hematologic malignancies, including *HLX*, *TFEC*, *GFI1*, *EVI2B*, *TLE4*, and *MYD88*, all shared with pediatric AML, suggest a transcriptional repressor activity for *MNX1* in AML affecting cell proliferation and myeloid differentiation. As pediatric AML with *MNX1* activation has a different activation event, does not have chr7q deletions or *BCOR* mutations, and is seen in infants at a different developmental state, the overlap of dysregulated key genes strongly connects the observed gene dysregulation to *MNX1* activity and not to confounding factors. We also identified a subgroup of *MNX1*-expressing cases without genomic rearrangements near *MNX1* which do not share the gene expression signature of the *MNX1*-rearranged cases. The expression of *MNX1* in these samples remains unexplained, but we observed that they have a very high frequency of *NPM1* mutations (67%), which might be linked to *MNX1* expression, as *NPM1* mutations have been shown to upregulate homeobox genes (58).

Taken together, our data suggest that the numerous genomic rearrangements in ckAML often lead to enhancer hijacking, a molecular event that may have been previously underestimated compared with oncofusions and CNAs. Understanding how the genes activated by this mechanism drive leukemia, or finding ways to stop this aberrant expression, could pave the way for personalized treatments targeting specific oncogenes.

## Methods

### Pyjacker Details

#### Identification of “Candidate Samples” with Breakpoints near a Gene

Only genes whose expression is greater than 1 transcript per million (TPM) in at least one sample are considered. For each gene, Pyjacker identifies “candidate samples” with a breakpoint near the gene and which may therefore overexpress this gene because of the rearrangement. Because promoter–enhancer interactions occur within TADs, Pyjacker selects samples which have a breakpoint in the same TAD as the gene. Any list of TADs can be provided, and in the present analysis, we used TADs derived from publicly available HiC data from HSPCs (Supplementary Table S17; ref. 19). To avoid missing events due to imprecise TAD boundaries, Pyjacker extends the TADs by 80 kb on each side. We note that this TAD extension did not impact the results on the ckAML cohort, as all reported events had breakpoints within the TAD of the activated gene, but it might improve the robustness in other cohorts. If a list of TADs is not provided as input, Pyjacker will instead consider all samples with breakpoints within a user-specified distance to the gene (1.5 Mb by default). All “candidate samples” for a particular gene will be scored to test whether these samples express this gene because of a structural rearrangement.

#### Overexpression Score

If a gene is activated by enhancer hijacking in a sample, we expect this sample to have a higher expression for this gene, compared with “reference samples” which do not have breakpoints near the gene. In order to remove the effect of amplifications and to focus on genes activated by enhancer hijacking, the expression values in TPM are corrected for copy number, if CNA data are provided: the expression values are multiplied by 2/(copy number). The expression values are then log-transformed: log(0.5+E). Then, Pyjacker computes the mean μ and SD σ of the gene expression in reference samples (which do not have breakpoints near the gene). For each candidate sample, Pyjacker computes the number of SDs away its expression lies from the mean, in which the SD is increased in order to avoid extreme scores when all reference samples have the same expression: t=(E-μ)/(σ+0.3) in which E is the expression of the gene in the candidate sample. This overexpression score is then transformed so that it is positive when the expression is more than two SDs above the mean and negative otherwise and to avoid very high or very low overexpression scores which would have a disproportionate effect on the final score: if t>2, $S_{overexpression}=\log\left(t-1\right)$, else $S_{overexpression}=-2\;\log\left(3-t\right)$.

### Allele-Specific Expression Score

If a gene is activated by enhancer hijacking, we would expect only the allele on the rearranged chromosome to be expressed, resulting in monoallelic expression. For each gene and each sample, heterozygous SNPs are identified in the WGS data, and if there is coverage in the RNA-seq, the numbers of reference and alternative reads in the RNA-seq data are counted. For each SNP, Pyjacker computes the log-likelihood ratio between monoallelic and biallelic expression. For monoallelic expression, we assume a mixture of two β-binomial distributions for the allelic read counts, with means centered on 2% and 98% (to account for possible low expression from the other allele). For biallelic expression, we assume a β-binomial distribution centered on 50%. The log-likelihood ratios from all SNPs in the gene are then combined to get the allele-specific expression (ASE) score, by averaging the log-likelihood ratios but still giving a higher score if several SNPs are present: $S_{ase}=\left(\overset{n}{\underset{i=0}{\sum }}{llr}_{i}\;\right)/\left(n+2\right)$ in which n is the number of SNPs in the gene. This score is positive if the allelic information supports a monoallelic expression, negative if it supports a biallelic expression, and close to 0 if it is unclear. We note that if no heterozygous SNPs are present in a gene in a sample, the ASE score will be 0, but this does not preclude the gene from being identified by Pyjacker if the overexpression and enhancer scores are positive. The ASE score is set to 0 for genes with copy number lower than two or greater than four, for genes on sex chromosomes, and for imprinted genes (if a list of imprinted genes is provided as input). If allelic read counts are not provided as input, Pyjacker can still be run and will in this case not use the ASE score, which will result in a higher FDR.

### Enhancer Score

A genomic rearrangement is more likely to result in enhancer hijacking if it brings a strong enhancer close to the target gene. Pyjacker can optionally take as input a list of enhancers, scored for enrichment of enhancer marks by ROSE (see section “Identification of myeloid enhancers” for the ChIP-seq data that we used in this study; refs. 66, 67). The list of enhancers provided must be derived from the same cell type as the cancer samples studied. If no enhancer data are available, the enhancer score will be set to 0.

Pyjacker identifies all enhancers which, after the rearrangement, likely come to the same TAD as the gene. This is done by considering the position and orientation of the breakpoints, but each breakpoint is considered independently, which might miss some enhancers in case of complex rearrangements with clustered breakpoints. Enhancers are ranked according to their enrichment, and Pyjacker computes the enhancer score by adding all scores but putting more weight on the strongest enhancers: $S_{enhancer}=\overset{n}{\underset{i=0}{\sum }}E_{i}/\left(i+1\right)$ in which n is the number of enhancers and $E_{i}$ is the enrichment for the i-th strongest enhancer.

### Combined Score

The overexpression, ASE, and enhancer scores are then combined with a weighted sum. Pyjacker also penalizes if the gene is deleted in the sample, because rearrangements leading to enhancer hijacking should not delete the activated gene. This results in a score for each pair of (gene, candidate sample):

$$S={\omega_{overexpression}S}_{overexpression}+{\omega_{ase}S}_{ase}\;+{\omega_{enhancer}S}_{enhancer}\;-\omega_{deletion}1_{deletion}$$

The weights can be set by the user, but their default values which should work well in all cases are $\omega_{overexpression}=4$, $\omega_{ase}=2$, $\omega_{enhancer}=1$, and $\omega_{deletion}=1$. $1_{deletion}$ is 1 if the gene is deleted in the sample and 0 otherwise.

### Aggregated Gene Score across Samples

In order to give more weight to genes which are activated in multiple samples, Pyjacker aggregates the scores from all samples for each gene:

$S_{gene}=5\overset{n}{\underset{i=0}{\sum }}S_{i}\;/\left(n+4\right)$ in which $S_{i}$ is the score from sample i.

### FDR

The gene scores reflect how likely a gene is to be activated by structural rearrangements in the cohort studied, but the values are somewhat arbitrary. In order to get a more interpretable FDR, Pyjacker computes a null distribution for these scores in the absence of enhancer hijacking. For each gene, the true “candidate samples” are excluded, and instead 1, 2, or 3 (number chosen randomly) random samples are chosen from the reference samples (without breakpoints near the gene) to be considered candidate samples and scored. This results in a list of null scores, in which only pairs of (gene, sample) without enhancer hijacking are used. The length of this list is equal to the number of genes ($n_{genes}$), so to increase the size of the list (and thus get more precise *P* values), this process is repeated $n_{iter}$ times ($n_{iter}=50$ by default), in which each time different random samples are selected for each gene, resulting in a list of $n_{iter}*n_{genes}$ null scores. This null distribution is used to compute an empirical *P* value for each gene. Finally, the Benjamini–Hochberg correction is used to correct for multiple testing, which results in an FDR.

### AML Cell Lines Used to Test Pyjacker

We tested Pyjacker using 10 AML cell lines: THP-1, LAMA-84, MONOMAC-1, MV-4-11, HEL92.1.7, EOL-1, OCI-AML3, GDM-1, MOLM-1, and MUTZ-3. Some of these cell lines had known enhancer hijacking events: *MECOM* in MOLM-1 and MUTZ-3 (16), *MNX1* in GDM-1 (31), and *MN1* in MUTZ-3 (32). WGS and RNA-seq data for THP-1, LAMA-84, MONOMAC-1, MV-4-11, HEL92.1.7, and EOL-1 were retrieved from the Cancer Cell Line Encyclopedia (68). RNA-seq and WGS data of GDM-1 were retrieved from Gene Expression Omnibus (GEO) accession GSE221753 and Sequence Read Archive (SRA) accession SRR23087016 (31). RNA-seq data of OCI-AML3 were retrieved from GEO accession GSE209777 (69). WGS data for OCI-AML3 and WGS and RNA-seq data for MOLM-1 and MUTZ-3 were performed for this study (see “Data Availability”). The sequencing data from cell lines were processed in the same way as patient samples (see below).

### Medulloblastoma Dataset

To evaluate the accuracy and efficacy of Pyjacker compared with cis-X, we applied them to a cohort of 120 medulloblastoma samples, which had been used by Northcott and colleagues (29, 30) to show that *GFI1* and *GFI1B* could be activated by enhancer hijacking in some cases of medulloblastoma.

### AML Patient Samples

The 39 ckAML samples were derived from a prospective clinical trial (NCT02348489) conducted in older, unfit, previously untreated patients with newly diagnosed AML (70). This clinical trial was conducted according to the Declaration of Helsinki, and written consent was obtained from the patients. Patient sex, age at diagnosis, and karyotype information are provided in Supplementary Table S6, but race, ethnicity, risk category, and disease stage were not available. Data on targeted DNA sequencing of this cohort and in part of EPIC BeadChip array analysis were previously reported by Jahn and colleagues (33). For this study, we selected 39 ckAML blood or BM samples (median age: 77 years), which had at least three CNAs detectable from the EPIC array data and for which sufficient material was still available for further profiling. Detailed patient characteristics, including sex, age, and cytogenetics, are provided in Supplementary Table S6.

### Generation and Processing of WGS Data

For both primary patient samples (blood or BM) and cell lines, DNA was isolated as previously described (19). The DNA was sequenced with NovaSeq 6000 S4, with read length of 2 × 150 bp and a coverage of 50× to 70× for each sample. The WGS data were aligned to the GRCh37 reference genome using Burrows-Wheeler Aligner-MEM [arXiv:1303.3997v2 (q-bio.GN)]. SVs were called with Manta (71), CNAs were called with Control-FREEC (72) and SNVs with Mutect2 (bioRxiv 10.1101/861054). Because no matched normal samples were available to identify somatic mutations, we only looked for SNVs in 52 genes known to be recurrently mutated in AML, as previously described (19). Chromothripsis was determined using ShatterSeek (73) using a criterion of at least 10 copy number switches in one chromosome. The WGS data processing, starting from the aligned BAM files, was done using a Nextflow workflow: https://github.com/CompEpigen/wf_WGS. All WGS plots were made using Figeno (74).

### RNA-seq

RNA was isolated as previously described (19). The RNA was sequenced with NovaSeq 6000 S2, with read length 2 × 101 bp and 180 to 250 million reads per sample. The RNA-seq data were processed using the nf-core rnaseq workflow v3.9, with alignment using STAR (75) and quantification using Salmon (76). Fusion transcripts were detected using Arriba (28). For ASE, we detected heterozygous SNPs in WGS data using HaplotypeCaller and used GATK ASEReadCounter to get allele-specific read counts in RNA-seq data at positions in which a heterozygous SNP was found. Differential gene expression analysis was run using the deseq2 (77) package v1.42.0 with log fold change shrinkage applied by the ashr (78) algorithm v2.2-63. Batch correction was applied for the MLL cohort following the generation of vst-transformed gene expression values for single gene expression visualization. The TARGET pediatric AML RNA-seq dataset was downloaded from UCSC XENA and analyzed using the same approach as the adult AML cohort. For cases with multiple sample points, primary specimens were selected over recurrent samples. BM samples were preferentially used over blood-derived samples, yielding overall two unique cases with the t(7;12)(q36;p13) karyotype. The IDs of the samples from the TARGET-AML cohort that were used, together with their t(7;12) status, origin (blood or BM), and recurrence, are provided in Supplementary Table S18. The Balgobind and colleagues (59) pediatric AML cohort and its corresponding GEO GSE17855 Affymetrix U133 Plus 2.0 microarray dataset was analyzed using the Limma (79) package v3.58.1 using the empirical Bayes algorithm with default settings. Cases with unknown karyotype were not considered.

### Validation of Breakpoints by Genomic PCR

PCR to confirm translocation t(1;3) in sample 16KM11270 was done with 10 cycles of touchdown from 59°C to 54.5°C, and 30 cycles at 54°C annealing temperature. PCR to confirm breakpoint 1 in sample 15PB8708 was done with 69°C annealing temperature for 35 cycles, and PCR for breakpoint 2 with 10 cycles of touchdown from 70°C to 65°C and 30 cycles at 65°C. Q5 High-Fidelity PCR Kit (New England Biolabs, #E0555) and, depending on the PCR reaction (primers in Supplementary Table S12), 20 to 160 ng genomic DNA were used. PCR products were analyzed on 1.2% Tris-borate, ethidium bromide–stained agarose gels, and gel images were recorded using a Bio-Rad Geldoc GO system (#12009077).

### scRNA-seq of Patients with del(7q) AML

scRNA-seq was performed for eight AML samples: four *MNX1*+ samples [three with del(7q) and one with an alternative rearrangement)] and four control *MNX1*− samples with del(7q). Only the *MNX1*+ sample with alternative rearrangement (15PB8708) was part of the initial 39 ckAML samples, the seven others come from other AML samples. Names and provenance (BM or PB) for these eight samples are provided in Supplementary Fig. S10. Cryopreserved samples from BM and PB were thawed at 37°C for 2 minutes before transferring to a 50-mL tube. Cells were diluted by adding incremental 1:1 volumes of DMEM/F12 media (Thermo Fisher Scientific) for 5 times with 1-minute wait in between each step. Cells were centrifuged at 300 rcf for 5 minutes and resuspended in 2 mL PBS (Thermo Fischer Scientific) + 0.04% BSA (Milteny Biotec). Libraries were generated using 20,000 single cells as input to the Chromium Controller using Chromium Next GEM Single-Cell 3′ Kit v3.1 (10× Genomics). From the single-cell sequencing libraries, we generated between 632 and 803 M (between 60,000 and 80,000 reads per cell) reads per sample using an Illumina NovaSeq 6000 S4 FlowCell. For processing (alignment to reference genome GRCh38, generation of count matrix) raw sequencing reads, Cell Ranger v7.1.0 was used. Subsequent analyses, including normalization (log-normalize), generation of a low-dimensional representation, and cluster annotation, were conducted using the Seurat v5 software package (80). Batch integration was performed with Canonical Correlation Analysis using Seurat’s IntegrateData function (81). For facilitating cluster annotation, we projected our data to the Triana and colleagues (61) reference atlas using scMap (82). We used Numbat (83) for inferring copy number losses and gains from the single-cell transcriptomic data. A cell was annotated as having del(7q) if the probability of the deletion as returned by Numbat was larger than 0.5.

### Identification of Myeloid Enhancers

We used public ChIP-seq data for H3K27ac and P300 from three myeloid cell lines: K562 [data from the ENCODE project (84), accessions ENCSR000AKP and ENCSR000EGE], MOLM-1 [data from ArrayExpress accession E-MTAB-2224 (16)], and Kasumi-1 (data from GEO accession GSE167163; bioRxiv 10.1101/2022.09.14.507850). We used ROSE (66, 67) to score and rank super-enhancers, in which transcription start sites were excluded. ROSE normally takes as input a single ChIP-seq experiment, but we found that the ranking was very variable depending on the dataset being used, so we used the six ChIP-seq datasets mentioned above and averaged the ROSE scores. The average ROSE scores were used as input to Pyjacker in order to compute the enhancer score.

### *MNX1* Expression Screen

For public cohorts profiled with RNA-seq, we considered a sample to be *MNX1*+ if its expression of *MNX1* was higher than 5 TPM, as it was 0 in most samples. Because most *MNX1*+ samples had expression values for *MNX1* greater than 100 TPM, we chose this threshold of 5 TPM to avoid noise from samples with very low *MNX1* expression.

For qRT-PCR, cDNA was generated from blood or BM AML samples with random hexamers and Superscript III reverse transcriptase (Invitrogen, #56575). Analysis (primers in Supplementary Table S12) was done with a primaQUANT CYBR mix (Steinbrenner Laborsyteme GmbH, #SL-9902) on a Roche LightCycler 480. Relative expression was determined with the 2^−Δ(Ct)^ method using Ct values of GAPDH or porphobilinogen deaminase (PBGD) for normalization. For each cohort, we computed the mean and SD for these values and considered samples to be *MNX1*+ if their values were higher than the mean plus three times the SD.

### PDX Model

PB samples from a patient with AML at first and second relapses were obtained from the Department of Internal Medicine III, Ludwig-Maximilians-Universität, Munich, Germany. Specimens were collected for diagnostic purposes. Written informed consent was obtained from the patient under the AMLCG Registry study (DRKS00020816). The study was performed in accordance with the ethical standards of the responsible committee on human experimentation (written approval by the Research Ethics Boards of the medical faculty of Ludwig-Maximilians-Universität, Munich, numbers 068-08 and 222-10) and with the Helsinki Declaration of 1975, as revised in 2013.

The PDX models AML-491 and AML-661 were established from primary patient cells at first and second relapses. The PDX cells harbored a del(7)(q21.13q36.3) and several AML-related mutations (Supplementary Table S19). Positive *MNX1* expression was determined via RNA-seq, and PDX cells were genetically modified as previously outlined in Zeller and colleagues (85). TAM-inducible shRNA constructs were generated as described in Carlet and colleagues (62) for two individual MNX1 shRNAs (76 & 82) and Renilla control shRNAs. Cre^ERT2^ and the shRNA cassettes were stably integrated into the AML-661 PDX model via lentiviral transduction using third-generation packaging plasmids [pMDLg/pRRE (Addgene#12251), pRSV-Rev (Addgene#12253), and the VSV-G envelope–expressing plasmid pMD2.G (Addgene#12259)] with the addition of polybrene (Sigma Aldrich, order no. H9268). Cre^ERT2^/shMNX1-76, Cre^ERT2^/shMNX1-82, Cre^ERT2^/shRenilla-1, and Cre^ERT2^/shRenilla-2 transgenic cells were enriched using a BD FACSAria III Cell Sorter (BD Biosciences) and serially transplanted into donor mice for amplification.

Animal trials were performed in accordance with the current ethical standards of the official committee on animal experimentation (written approval by Regierung von Oberbayern, tierversuche@reg-ob.bayern.de; ROB-55.2Vet-2532.Vet_02-16-7 and ROB-55.2-2532.Vet_02-20-159). In general, PDX cells were amplified in 10- to 26-week-old male or female NOD.Cg-*Prkdc*^*scid*^*Il2rg*^*tm1Wjl*^/SzJ mice (The Jackson Laboratory). Mice were kept in animal rooms of the Laboratory Animal Breeding and Husbandry Unit of Helmholtz Zentrum München under specified pathogen-free conditions with a 12/12-hour light cycle. The animal rooms of the barriers were fully air-conditioned with a temperature of 20°C to 24°C and 45% to 65% humidity according to Annex A of the European Convention 2007/526 EC. The maximum stocking density of the cages corresponds to Annex III of the 2010/63 European Union. The cages were constantly filled with structural enrichment, and the animals had unlimited access to food and water. During the experiment, mice were kept in individually ventilated cages (IVC). The cages were only opened one at a time at a cage-changing station, and the experimenter’s gloves were disinfected with disinfectant each time before a mouse was removed from the cage. Hygiene monitoring was carried out at least quarterly in accordance with the current Federation of European Laboratory Animal Science Associations (FELASA) recommendation: In the animal housing areas equipped with IVC systems, exhaust dust from the IVC ventilation units was tested for all FELASA-listed pathogens by PCR.

### 4C

About 2 million cells per sample were used for 4C according to van de Werken and colleagues (86). Two rounds of restriction digestion/T4 DNA ligation were applied, using *Bgl*II in combination with *Nla*III. In a first PCR step, second ligation products, inverse primers (Supplementary Table S20), and Q5 high-fidelity enzyme (New England Biolabs, #M0491) were used with reaction conditions at 98°C for 30 seconds; 10 cycles with 98°C for 15 seconds; 63°C, 57°C, or 54°C, depending on the viewpoint, for 20 seconds with 0.5°C touchdown per cycle; 72°C for 2 minutes; 30 or 25 cycles with 98°C for 15 seconds; 58°C, 52°C, or 49°C, depending on the viewpoint, for 20 seconds; and 72°C for 2 minutes, finally followed by 72°C for 1 minute. Purification of PCR products, generation of sequencing libraries, and sequencing were done as described previously (31). PCR products were purified with HighPrep beads (Biozym, cat. no. 220002), and their concentrations were determined by Qubit dsDNA HS Assay (Thermo Fisher Scientific, cat. no. Q32854). The sequencing libraries were generated with about 5 ng purified PCR products by RT-PCR to monitor amplification progress using a LightCycler 480 (Roche) and 25 μL reaction volumes using KAPA2G Robust HotStart ReadyMix (Merck, cat. no. KK5702) at 95°C for 3 minutes (initial melting) and 95°C for 20 seconds, 62°C for 15 seconds, and 72°C for 40 seconds (cycling). Each 0.75 μL of primers (stock concentration 10 μmol/L) Tn5mCP1n (AATGATACGGCGACCACCGAGATCTACACTCGTCGGCAGCGTC) and Tn5mCBar (CAAGCAGAAGACGGCATACGAGAT[barcode]GTCTCGTGGGCTCGG) were used. Sequencing libraries resulting from PCR products were bead-purified, DNA concentration was determined by Qubit dsDNA HS Assay, and product sizes were determined by TapeStation 4150 analysis with D1000 High Sensitivity Assay (Agilent, cat. no. 5067- 5585). Sequencing libraries were pooled in equimolar ratios and analyzed on a NextSeq 550 machine (Illumina), midoutput, 75 PE mode.

### ACT-seq

Genome-wide targeting of histone modifications was done by ACT-seq according to Carter and colleagues (87) with some modifications using a self-prepared pA-Tn5ase protein (31) and using the antibodies listed in Supplementary Table S21. To generate a pA-Tn5 transposome (pA-Tn5ome), pA-Tn5ase and Tn5ME-A+B load adaptor were mixed such that both components had a concentration of 3.3 μmol/L in complex formation buffer (CB). pA-Tn5ome–antibody (pA-Tn5ome–ab) complexes were generated by mixing 1 μL pA-Tn5ome with 0.8 μL CB and 0.8 μL antibody solution. Per tagmentation and pA-Tn5ome–ab complex binding, 50,000 cells were used. For normalization of sequence reads between biological replicates, about 4,000 permeabilized nuclei of yeast *Saccharomyces cerevisiae*, prepared according to (88) and incubated with pA-Tn5ome–ab complex targeting yeast H2B, were spiked into each pA-Tn5ome–ab complex/cell mix. Tagmentation at 37°C for 30 minutes was started by addition of 10 μmol/L MgCl_2_ (final concentration) followed by a 30-minute proteinase K (20 μg; Qiagen, #19133) treatment at 55°C. DNA was purified using MinElute Kit (Qiagen, #28004) and eluted with 20 μL elution buffer. Sequencing libraries were generated under real-time conditions to monitor amplification progress using a LightCycler 480 in 50 μL reaction mixes consisting of 20 μL tagmented DNA eluate, 25 μL NEBNext High-Fidelity 2× Mix (New England Biolabs, cat. no. M0541), 0.5 μL 100xSYBRGreen, and each 2.5 μL primer Tn5McP1n and Tn5mCBar (stock concentration 10 μmol/L, see above). Reaction conditions were 72°C for 5 minutes; 98°C, 30 seconds; cycling with 98°C, 10 seconds; 63°C, 10 seconds; and 72°C, 10 seconds. PCR products were purified with HighPrep beads. DNA concentration and fragment size were determined as described above. Six to eight differently barcoded libraries were multiplexed and sequenced as described above on a NextSeq 550 system.

### ATAC-seq

ATAC-seq was done essentially as described by Corces and colleagues (89) using about 50,000 cells and the Nextera DNA Library Prep Kit (Illumina, #15028212). In brief, cells were lysed in ATAC-RSB buffer containing 0.5 μL NP40 10%, 0.5 μL Tween-20 10%, and 0.5 μL digitonin 1% followed by short incubation in ATAC-RSB containing 0.1% Tween-20. Tagmentation was done in a 50 μL mix at 37°C for 30 minutes in a thermomixer (Eppendorf, comfort 5355) with 1,000 rpm. Reactions were stopped by addition of 20 μL 5 mol/L guanidinium thiocyanate, and DNA was purified with 140 μL HighPrep beads. Libraries were generated under real-time conditions and processed as described for ACT-seq, but cycling conditions were 98°C for 10 seconds; 63°C, 30 seconds; and 72°C, 30 seconds.

### 4C Sequencing, ACT-seq, and ATAC-seq Data Analysis

4C sequencing data processing and analysis were done with the pipe4C pipeline (90) using single reads starting with a *Bgl*II site containing viewpoint primer; the pipe4C pipeline was applied with default parameters under R v3.6.2. ACT-seq and ATAC-seq data were analyzed as described previously (31). Upstream processing of ATAC-seq and ACT-seq data was performed using TrimGalore v0.4.4 (RRID: SCR_011847) together with Cutadapt v1.14 (RRID: SCR_011841) applying the nondefault parameters “—paired,” “—nextera,” “—length_1 35,” and “—length_2 35” to perform adapter and quality trimming. Bowtie2 v2.2.6 (RRID: SCR_016368) was used with the “—very sensitive” flag and a maximum insertion length of 2,500 bp to map trimmed reads against the GRCh37/hg19 reference genome. Aligned reads belonging to the same lane-multiplexed library were combined using SAMtools merge v1.5 (RRID: SCR_006525). PCR duplicates were removed by means of Picard MarkDuplicates v2.17.4 for ATAC-seq but not ACT-seq data. Discordant mappings and alignments with a Phred score below 20 were removed using SAMtools view. Trimmed ACT-seq reads were additionally aligned against the *Saccharomyces cerevisiae* R64 reference genome and post-aligned as described above. To derive a library-specific scaling factor, the multiplicative inverse of the number of filtered alignments against the yeast genome was calculated. This normalization leads to signal ranges in BigWig files and Integrative Genomics Viewer browser tracks close to zero. Coverage tracks were generated using the bamCoverage functionality of deepTools v3.1.1 (RRID: SCR_016366) with the nondefault parameters “—ignoreForNormalization chrM chrY chrX” and “—effectiveGenomeSize 2652783500” as well as the “—scaleRatio” option to specify the spike-in–derived scaling factor. ATAC-seq accessibility signals were smoothed by centering a 73 bp window on the transposition event’s midpoint of each read using a custom script; the resulting tag coordinates were used for all downstream analyses. The analysis procedures were implemented as fully containerized workflows using the Common Workflow Language v1.0. BigWig tracks were visualized using Figeno (74).

### CRISPR/Cas9-Mediated Enhancer Insertion

A 1 kb region (chr7:92384001–92385000, GRCh37/hg19) containing a putative enhancer was inserted upstream of the *MNX1* promoter (chr7:156816239, GRCh37/hg19) in ChiPSC22 (Takara Bio Europe) by CRISPR/Cas9 editing as previously described (91). In short, ChiPSC22 cells were nucleofected with the Cas9 ribonucleoprotein complex and a homology-directed repair (HDR) donor template containing the putative enhancer sequence and 200 bp homology arms on each site. The CRISPR RNA was designed using the Alt-R Custom Cas9 crRNA Design Tool (Integrated DNA Technologies), and the HDR donor template was ordered as double-strand DNA HDR Donor Block (Integrated DNA Technologies). Per 20 μL transfection, 500 ng of the HDR Donor Block were used. Clones with successful integration of the enhancer on one allele were selected by PCR using the following primers: AAAAGGACATGGGGATGCGT and GAAGCTGATCTTCCCTGAGGTT. Two cell lines were validated using WGS. Cell lines were differentiated to HSPCs as previously described (91). RNA was isolated from HSPCs using RNeasy Plus Mini Kit (Qiagen) and sequenced as described above.

### Competitive *MNX1* Knockdown *In Vivo* Assays

#### Constitutive Knockdown

Transgenic AML PDX cells were isolated from BM of donor mice and cultured in StemPro-34 medium (Thermo Fisher Scientific) with penicillin/streptomycin, L-glutamine (both Thermo Fisher Scientific), 10 ng/mL hrFLT3L (R&D Systems), 10 ng/mL hrSCF, 10 ng/mL hrTPO, and 10 ng/mL hrIL3 (all Peprotech; ref. 92) at a density of 10^6^ cells/mL at 37°C, 5% CO_2_. For *ex vivo* flipping of the shRNA cassettes, the cells were treated using 200 nmol/L (Z)-4-hydroxytamoxifen (Sigma Aldrich, #H7904). This induces flipping of the shRNA cassette, which leads to the expression of the respective shRNA and a switch of the expressed fluorochrome from mTagBFP to eGFP and from iRFP720 to T-Sapphire, respectively. Cells harboring the flipped cassette were enriched via FACS. MNX1 shRNA– and Renilla control shRNA–expressing cells were mixed in a 1:1 ratio and injected into three mice per MNX1 shRNA via tail vein injection (1 × 10^6^ cells per population, 2 × 10^6^ per mouse). The individual input mixes were measured by flow cytometry for each animal before injection as an input sample (Supplementary Fig. S12A–S12E). Outgrowth of tumor cells was monitored by repeated blood samplings and staining for hCD33^+^ cells (BD Pharmingen PE Mouse Anti-Human CD33, clone WM53, cat. no. 555450; RRID: AB_395843). At an advanced stage of leukemia (hCD33^+^ cells >60%), mice were sacrificed and PDX cells were isolated from the BM, spleen, and blood.

#### Inducible Knockdown

*In vivo* induction of the MNX1 shRNA expression was performed according to Carlet and colleagues (62). Transgenic AML PDX cells were isolated from BM of donor mice. Cre^ERT2^/shMNX1 and Cre^ERT2^/shRenilla transgenic cells were mixed in a 1:1 ratio and injected into mice via tail vein injection (*N* = 13; 1 × 10^6^ cells per population and mouse). Fifty mg/kg TAM (Sigma Aldrich, #T5648) was administered once 14 days after transplantation via oral gavage as previously described. Mice were sacrificed on the day of TAM administration without receiving TAM, 3 days after TAM administration, and at an advanced stage of leukemia (hCD33^+^ cells >60%).

### Statistical Analyses

The FDR for Pyjacker was computed by converting the scores into empirical *P* values and correcting for multiple testing, as described above. For the analysis of the MNX1 *in vivo* knockdown, we compare the ratio of the two flipped cell populations by performing two-tailed unpaired *t* tests using Prism 10 (GraphPad Prism).

### Data Availability

WGS and RNA-seq data of patient samples are available at the European Genome-phenome Archive under the accession EGAS50000000743. All preprocessed data used as input to Pyjacker for the ckAML cohort are provided in the GitHub repository at https://github.com/CompEpigen/pyjacker/tree/main/data. WGS data of the cell line OCI-AML3 and WGS and RNA-seq data of the cell lines MOLM-1 and MUTZ-3 were uploaded to the SRA under project PRJNA1140384. The source code for Pyjacker is available at https://github.com/CompEpigen/pyjacker. This manuscript describes pyjacker version 1.1.2, which is archived at Zenodo https://doi.org/10.5281/zenodo.14516090. A Code Ocean capsule reproducing Pyjacker’s results on the ckAML dataset is also available at https://codeocean.com/capsule/6670298/tree/v2. The Nextflow workflow used to prepare Pyjacker’s inputs, starting from BAM files, is available at https://github.com/CompEpigen/wf_WGS.



## Supplementary Material

Supplementary Tables ST1 Methods for enhancer hijacking detection ST2 Ranked and scored hematopoietic enhancers (based on ChIP-seq data for H3K27ac and P300 from K562, MOLM1 and Kasumi1) ST3 Pyjacker results for the 10 AML cell lines ST4 Pyjacker results for the 120 medulloblastoma samples ST5 Cis-x results for the 120 medulloblastoma samples ST6 Summary information for the 39 ckAML patient samples ST7 SNVs detected in WGS data for the 39 ckAML samples ST8 SVs detected in WGS data for the 39 ckAML samples ST9 CNAs detected in WGS data for the 39 ckAML samples ST10 Fusion transcripts detected in RNAseq data for the 39 ckAML samples ST11 Pyjacker results for the 39 ckAML samples ST12 Primers for qRT-PCR and validation of breakpoints (hg19) ST13 Summary of the MNX1 expression screens ST14 Summary of the MNX1-expressing samples profiled with WGS ST15 Mutations detected in the MNX1-expressing samples profiled with WGS ST16 Results of the differential expression analysis (adult and pediatric AML) ST17 TADs derived from HiC data of HSPCs ST18 Samples IDs from the TARGET-AML cohort used for the RNA-seq analysis ST19 Clinical characteristics of the patient samples from which were derived the PDX ST20 Primers and conditions used for 4C ST21 Antibodies used

Supplementary Figures Supplementary Figure 1. Summary of the somatic alterations in the 39 ckAML samples. Supplementary Figure 2. Proportion of samples expressing the top pyjacker hits, for several AML cohorts profiled with RNA-seq. Supplementary Figure 3. Example rearrangements leading to gene activation and TP53 inactivation. Supplementary Figure 4. Rearrangements leading to MECOM monoallelic expression in sample 15KM20146. Supplementary Figure 5. Validation of the translocation t(1;3)(p36;q21) in sample 16KM11270 and of the breakpoints for the CDK6 enhancer duplication next to MNX1 in sample 15PB8708 by genomic PCR. Supplementary Figure 6. Deletion between the TCR beta locus and MNX1 leading to MNX1 expression. Supplementary Figure 7. Alternative rearrangements leading to MNX1 expression. Supplementary Figure 8. Differential gene expression analysis of MNX1 status in adult and pediatric AML. Supplementary Figure 9. Gene expressions of 25 selected cancer and hematological development associated genes differentially expressed under MNX1 activation. Supplementary Figure 10. Copy number alterations on chromosome 7 for samples profiled with scRNA-seq. Supplementary Figure 11. Reciprocal 4C. Supplementary Figure 12: Knockdown of MNX1 reduces tumor load of AML PDX cells in vivo.

## References

[1] Cancer Genome Atlas Research Network; Ley TJ, Miller C, Ding L, Raphael BJ, Mungall AJ, Robertson A, . Genomic and epigenomic landscapes of adult de novo acute myeloid leukemia. N Engl J Med 2013;368:2059–74. 23634996 10.1056/NEJMoa1301689 PMC3767041

[2] Papaemmanuil E , Gerstung M, Bullinger L, Gaidzik VI, Paschka P, Roberts ND, . Genomic classification and prognosis in acute myeloid leukemia. N Engl J Med 2016;374:2209–21. 27276561 10.1056/NEJMoa1516192 PMC4979995

[3] Arber DA , Orazi A, Hasserjian RP, Borowitz MJ, Calvo KR, Kvasnicka H-M, . International consensus classification of myeloid neoplasms and acute leukemias: integrating morphologic, clinical, and genomic data. Blood 2022;140:1200–28. 35767897 10.1182/blood.2022015850 PMC9479031

[4] Mrózek K . Cytogenetic, molecular genetic, and clinical characteristics of acute myeloid leukemia with a complex karyotype. Semin Oncol 2008;35:365–77. 18692687 10.1053/j.seminoncol.2008.04.007 PMC3640813

[5] Korbel JO , Campbell PJ. Criteria for inference of chromothripsis in cancer genomes. Cell 2013;152:1226–36. 23498933 10.1016/j.cell.2013.02.023

[6] Rode A , Maass KK, Willmund KV, Lichter P, Ernst A. Chromothripsis in cancer cells: an update. Int J Cancer 2016;138:2322–33. 26455580 10.1002/ijc.29888

[7] Fontana MC , Marconi G, Feenstra JDM, Fonzi E, Papayannidis C, Ghelli Luserna di Rorá A, . Chromothripsis in acute myeloid leukemia: biological features and impact on survival. Leukemia 2018;32:1609–20. 29472722 10.1038/s41375-018-0035-y PMC6035145

[8] Schoch C , Haferlach T, Bursch S, Gerstner D, Schnittger S, Dugas M, . Loss of genetic material is more common than gain in acute myeloid leukemia with complex aberrant karyotype: a detailed analysis of 125 cases using conventional chromosome analysis and fluorescence in situ hybridization including 24-color FISH. Genes Chromosomes Cancer 2002;35:20–9. 12203786 10.1002/gcc.10088

[9] Rücker FG , Bullinger L, Schwaenen C, Lipka DB, Wessendorf S, Fröhling S, . Disclosure of candidate genes in acute myeloid leukemia with complex karyotypes using microarray-based molecular characterization. J Clin Oncol 2006;24:3887–94. 16864856 10.1200/JCO.2005.04.5450

[10] Schoch C , Kern W, Kohlmann A, Hiddemann W, Schnittger S, Haferlach T. Acute myeloid leukemia with a complex aberrant karyotype is a distinct biological entity characterized by genomic imbalances and a specific gene expression profile. Genes Chromosomes Cancer 2005;43:227–38. 15846790 10.1002/gcc.20193

[11] Halik A , Tilgner M, Silva P, Estrada N, Altwasser R, Jahn E, . Genomic characterization of AML with aberrations of chromosome 7: a multinational cohort of 519 patients. J Hematol Oncol 2024;17:70. 39160538 10.1186/s13045-024-01590-1 PMC11331663

[12] Greenberg P , Cox C, LeBeau MM, Fenaux P, Morel P, Sanz G, . International scoring system for evaluating prognosis in myelodysplastic syndromes. Blood 1997;89:2079–88. 9058730

[13] Inaba T , Honda H, Matsui H. The enigma of monosomy 7. Blood 2018;131:2891–8. 29615405 10.1182/blood-2017-12-822262

[14] McNerney ME , Brown CD, Wang X, Bartom ET, Karmakar S, Bandlamudi C, . *CUX1* is a haploinsufficient tumor suppressor gene on chromosome 7 frequently inactivated in acute myeloid leukemia. Blood 2013;121:975–83. 23212519 10.1182/blood-2012-04-426965 PMC3567344

[15] Chen C , Liu Y, Rappaport AR, Kitzing T, Schultz N, Zhao Z, . *MLL3* is a haploinsufficient 7q tumor suppressor in acute myeloid leukemia. Cancer Cell 2014;25:652–65. 24794707 10.1016/j.ccr.2014.03.016 PMC4206212

[16] Gröschel S , Sanders MA, Hoogenboezem R, de Wit E, Bouwman BAM, Erpelinck C, . A single oncogenic enhancer rearrangement causes concomitant *EVI1* and *GATA2* deregulation in leukemia. Cell 2014;157:369–81. 24703711 10.1016/j.cell.2014.02.019

[17] Montefiori LE , Bendig S, Gu Z, Chen X, Pölönen P, Ma X, . Enhancer hijacking drives oncogenic BCL11B expression in lineage-ambiguous stem cell leukemia. Cancer Discov 2021;11:2846–67. 34103329 10.1158/2159-8290.CD-21-0145 PMC8563395

[18] von Bergh ARM , van Drunen E, van Wering ER, van Zutven LJCM, Hainmann I, Lönnerholm G, . High incidence of t(7;12)(q36;p13) in infant AML but not in infant ALL, with a dismal outcome and ectopic expression of HLXB9. Genes Chromosomes Cancer 2006;45:731–9. 16646086 10.1002/gcc.20335

[19] Weichenhan D , Riedel A, Sollier E, Toprak UH, Hey J, Breuer K, . Altered enhancer-promoter interaction leads to MNX1 expression in pediatric acute myeloid leukemia with t(7;12)(q36;p13). Blood Adv 2024;8:5100–11. 39121370 10.1182/bloodadvances.2023012161 PMC11460460

[20] Weischenfeldt J , Dubash T, Drainas AP, Mardin BR, Chen Y, Stütz AM, . Pan-cancer analysis of somatic copy-number alterations implicates IRS4 and IGF2 in enhancer hijacking. Nat Genet 2017;49:65–74. 27869826 10.1038/ng.3722 PMC5791882

[21] Zhang Y , Chen F, Creighton CJ. SVExpress: identifying gene features altered recurrently in expression with nearby structural variant breakpoints. BMC Bioinformatics 2021;22:135. 33743584 10.1186/s12859-021-04072-0 PMC7981925

[22] Yu A , Yesilkanal AE, Thakur A, Wang F, Yang Y, Phillips W, . HYENA detects oncogenes activated by distal enhancers in cancer. Nucleic Acids Res 2024;52:e77. 39051548 10.1093/nar/gkae646 PMC11381332

[23] Liu Y , Li C, Shen S, Chen X, Szlachta K, Edmonson MN, . Discovery of regulatory noncoding variants in individual cancer genomes by using cis-X. Nat Genet 2020;52:811–8. 32632335 10.1038/s41588-020-0659-5 PMC7679232

[24] Wang X , Xu J, Zhang B, Hou Y, Song F, Lyu H, . Genome-wide detection of enhancer-hijacking events from chromatin interaction data in rearranged genomes. Nat Methods 2021;18:661–8. 34092790 10.1038/s41592-021-01164-w PMC8191102

[25] Creyghton MP , Cheng AW, Welstead GG, Kooistra T, Carey BW, Steine EJ, . Histone H3K27ac separates active from poised enhancers and predicts developmental state. Proc Natl Acad Sci U S A 2010;107:21931–6. 21106759 10.1073/pnas.1016071107 PMC3003124

[26] Visel A , Blow MJ, Li Z, Zhang T, Akiyama JA, Holt A, . ChIP-seq accurately predicts tissue-specific activity of enhancers. Nature 2009;457:854–8. 19212405 10.1038/nature07730 PMC2745234

[27] Haas BJ , Dobin A, Li B, Stransky N, Pochet N, Regev A. Accuracy assessment of fusion transcript detection via read-mapping and de novo fusion transcript assembly-based methods. Genome Biol 2019;20:213. 31639029 10.1186/s13059-019-1842-9 PMC6802306

[28] Uhrig S , Ellermann J, Walther T, Burkhardt P, Fröhlich M, Hutter B, . Accurate and efficient detection of gene fusions from RNA sequencing data. Genome Res 2021;31:448–60. 33441414 10.1101/gr.257246.119 PMC7919457

[29] Northcott PA , Lee C, Zichner T, Stütz AM, Erkek S, Kawauchi D, . Enhancer hijacking activates GFI1 family oncogenes in medulloblastoma. Nature 2014;511:428–34. 25043047 10.1038/nature13379 PMC4201514

[30] Northcott PA , Buchhalter I, Morrissy AS, Hovestadt V, Weischenfeldt J, Ehrenberger T, . The whole-genome landscape of medulloblastoma subtypes. Nature 2017;547:311–7. 28726821 10.1038/nature22973 PMC5905700

[31] Weichenhan D , Riedel A, Meinen C, Basic A, Toth R, Bähr M, . Translocation t(6;7) in AML-M4 cell line GDM-1 results in *MNX1* activation through enhancer-hijacking. Leukemia 2023;37:1147–50. 36949154 10.1038/s41375-023-01865-5 PMC10169647

[32] Riedel SS , Lu C, Xie HM, Nestler K, Vermunt MW, Lenard A, . Intrinsically disordered Meningioma-1 stabilizes the BAF complex to cause AML. Mol Cell 2021;81:2332–48.e9. 33974912 10.1016/j.molcel.2021.04.014 PMC8380056

[33] Jahn E , Saadati M, Fenaux P, Gobbi M, Roboz GJ, Bullinger L, . Clinical impact of the genomic landscape and leukemogenic trajectories in non-intensively treated elderly acute myeloid leukemia patients. Leukemia 2023;37:2187–96. 37591941 10.1038/s41375-023-01999-6 PMC10624608

[34] Rücker FG , Schlenk RF, Bullinger L, Kayser S, Teleanu V, Kett H, . *TP53* alterations in acute myeloid leukemia with complex karyotype correlate with specific copy number alterations, monosomal karyotype, and dismal outcome. Blood 2012;119:2114–21. 22186996 10.1182/blood-2011-08-375758

[35] Bottomly D , Long N, Schultz AR, Kurtz SE, Tognon CE, Johnson K, . Integrative analysis of drug response and clinical outcome in acute myeloid leukemia. Cancer Cell 2022;40:850–64.e9. 35868306 10.1016/j.ccell.2022.07.002 PMC9378589

[36] Bolouri H , Farrar JE, Triche T, Ries RE, Lim EL, Alonzo TA, . The molecular landscape of pediatric acute myeloid leukemia reveals recurrent structural alterations and age-specific mutational interactions. Nat Med 2018;24:103–12. 29227476 10.1038/nm.4439 PMC5907936

[37] Mochizuki N , Shimizu S, Nagasawa T, Tanaka H, Taniwaki M, Yokota J, . A novel gene, *MEL1*, mapped to 1p36.3 is highly homologous to the *MDS1/EVI1* gene and is transcriptionally activated in t(1;3)(p36;q21)-positive leukemia cells. Blood 2000;96:3209–14. 11050005

[38] Pinheiro I , Margueron R, Shukeir N, Eisold M, Fritzsch C, Richter FM, . Prdm3 and Prdm16 are H3K9me1 methyltransferases required for mammalian heterochromatin integrity. Cell 2012;150:948–60. 22939622 10.1016/j.cell.2012.06.048

[39] List J , Sollier E, Brown-Burke F, Kelly K, Pfeifer D, Shlyakhto V, . Genocopy of EVI1-AML with paraneoplastic diabetes insipidus: overexpression by t(1;2)(p36;p21) and enhancer hijacking. Br J Haematol 2024;00:1–5. 10.1111/bjh.19922 PMC12916193 39587921

[40] Gröschel S , Lugthart S, Schlenk RF, Valk PJM, Eiwen K, Goudswaard C, . High EVI1 expression predicts outcome in younger adult patients with acute myeloid leukemia and is associated with distinct cytogenetic abnormalities. J Clin Oncol 2010;28:2101–7. 20308656 10.1200/JCO.2009.26.0646

[41] Howard JC , Berger L, Bani MR, Hawley RG, Ben-David Y. Activation of the erythropoietin gene in the majority of F-MuLV-induced erythroleukemias results in growth factor independence and enhanced tumorigenicity. Oncogene 1996;12:1405–15. 8622856

[42] Chrétien S , Duprez V, Maouche L, Gisselbrecht S, Mayeux P, Lacombe C. Abnormal erythropoietin (Epo) gene expression in the murine erythroleukemia IW32 cells results from a rearrangement between the G-protein β2 subunit gene and the Epo gene. Oncogene 1997;15:1995–9. 9365246 10.1038/sj.onc.1201364

[43] Klingmüller U , Bergelson S, Hsiao JG, Lodish HF. Multiple tyrosine residues in the cytosolic domain of the erythropoietin receptor promote activation of STAT5. Proc Natl Acad Sci U S A 1996;93:8324–8. 8710869 10.1073/pnas.93.16.8324 PMC38669

[44] Richmond TD , Chohan M, Barber DL. Turning cells red: signal transduction mediated by erythropoietin. Trends Cell Biol 2005;15:146–55. 15752978 10.1016/j.tcb.2005.01.007

[45] Jayavelu AK , Schnöder TM, Perner F, Herzog C, Meiler A, Krishnamoorthy G, . Splicing factor YBX1 mediates persistence of JAK2-mutated neoplasms. Nature 2020;588:157–63. 33239784 10.1038/s41586-020-2968-3

[46] Perner F , Perner C, Ernst T, Heidel FH. Roles of JAK2 in aging, inflammation, hematopoiesis and malignant transformation. Cells 2019;8:854. 31398915 10.3390/cells8080854 PMC6721738

[47] Iacobucci I , Wen J, Meggendorfer M, Choi JK, Shi L, Pounds SB, . Genomic subtyping and therapeutic targeting of acute erythroleukemia. Nat Genet 2019;51:694–704. 30926971 10.1038/s41588-019-0375-1 PMC6828160

[48] Fagnan A , Bagger FO, Piqué-Borràs M-R, Ignacimouttou C, Caulier A, Lopez CK, . Human erythroleukemia genetics and transcriptomes identify master transcription factors as functional disease drivers. Blood 2020;136:698–714. 32350520 10.1182/blood.2019003062 PMC8215330

[49] Takeda J , Yoshida K, Nakagawa MM, Nannya Y, Yoda A, Saiki R, . Amplified *EPOR/JAK2* genes define a unique subtype of acute erythroid leukemia. Blood Cancer Discov 2022;3:410–27. 35839275 10.1158/2643-3230.BCD-21-0192 PMC9894574

[50] Andersson LC , Nilsson K, Gahmberg CG. K562—a human erythroleukemic cell line. Int J Cancer 1979;23:143–7. 367973 10.1002/ijc.2910230202

[51] Lo AWI , Sabatier L, Fouladi B, Pottier G, Ricoul M, Murnane JP. DNA amplification by breakage/fusion/bridge cycles initiated by spontaneous telomere loss in a human cancer cell line. Neoplasia 2002;4:531–8. 12407447 10.1038/sj.neo.7900267 PMC1503667

[52] Iacobucci I , Li Y, Roberts KG, Dobson SM, Kim JC, Payne-Turner D, . Truncating erythropoietin receptor rearrangements in acute lymphoblastic leukemia. Cancer Cell 2016;29:186–200. 26859458 10.1016/j.ccell.2015.12.013 PMC4750652

[53] Khan I , Amin MA, Eklund EA, Gartel AL. Regulation of HOX gene expression in AML. Blood Cancer J 2024;14:42. 38453907 10.1038/s41408-024-01004-y PMC10920644

[54] Cools J , Mentens N, Odero MD, Peeters P, Wlodarska I, Delforge M, . Evidence for position effects as a variant ETV6-mediated leukemogenic mechanism in myeloid leukemias with a t(4;12)(q11-q12;p13) or t(5;12)(q31;p13). Blood 2002;99:1776–84. 11861295 10.1182/blood.v99.5.1776

[55] Müller-Jochim A , Meggendorfer M, Walter W, Haferlach T, Kern W, Haferlach C. AML with t(4;12)(q12;p13): a detailed genomic and transcriptomic analysis reveals genomic breakpoint heterogeneity, absence of *PDGFRA* fusion transcripts and presence of *PDGFRA* overexpression in a subset of cases. Blood 2023;142:6014.

[56] Ottema S , Mulet-Lazaro R, Beverloo HB, Erpelinck C, van Herk S, van der Helm R, . Atypical 3q26/MECOM rearrangements genocopy inv(3)/t(3;3) in acute myeloid leukemia. Blood 2020;136:224–34. 32219447 10.1182/blood.2019003701

[57] Höllein A , Twardziok SO, Walter W, Hutter S, Baer C, Hernandez-Sanchez JM, . The combination of WGS and RNA-seq is superior to conventional diagnostic tests in multiple myeloma: ready for prime time? Cancer Genet 2020;242:15–24. 31980417 10.1016/j.cancergen.2020.01.001

[58] Brunetti L , Gundry MC, Sorcini D, Guzman AG, Huang Y-H, Ramabadran R, . Mutant NPM1 maintains the leukemic state through HOX expression. Cancer Cell 2018;34:499–512.e9. 30205049 10.1016/j.ccell.2018.08.005 PMC6159911

[59] Balgobind BV , Van den Heuvel-Eibrink MM, De Menezes RX, Reinhardt D, Hollink IHIM, Arentsen-Peters STJCM, . Evaluation of gene expression signatures predictive of cytogenetic and molecular subtypes of pediatric acute myeloid leukemia. Haematologica 2011;96:221–30. 20971820 10.3324/haematol.2010.029660 PMC3031689

[60] Ragusa D , Cicirò Y, Federico C, Saccone S, Bruno F, Saeedi R, . Engineered model of t(7;12)(q36;p13) AML recapitulates patient-specific features and gene expression profiles. Oncogenesis 2022;11:50. 36057683 10.1038/s41389-022-00426-2 PMC9440899

[61] Triana S , Vonficht D, Jopp-Saile L, Raffel S, Lutz R, Leonce D, . Single-cell proteo-genomic reference maps of the hematopoietic system enable the purification and massive profiling of precisely defined cell states. Nat Immunol 2021;22:1577–89. 34811546 10.1038/s41590-021-01059-0 PMC8642243

[62] Carlet M , Völse K, Vergalli J, Becker M, Herold T, Arner A, . In vivo inducible reverse genetics in patients’ tumors to identify individual therapeutic targets. Nat Commun 2021;12:5655. 34580292 10.1038/s41467-021-25963-z PMC8476619

[63] Xu J , Song F, Lyu H, Kobayashi M, Zhang B, Zhao Z, . Subtype-specific 3D genome alteration in acute myeloid leukaemia. Nature 2022;611:387–98. 36289338 10.1038/s41586-022-05365-x PMC10060167

[64] Federico C , Owoka T, Ragusa D, Sturiale V, Caponnetto D, Leotta CG, . Deletions of chromosome 7q affect nuclear organization and *HLXB9* gene expression in hematological disorders. Cancers 2019;11:585. 31027247 10.3390/cancers11040585 PMC6521283

[65] Waraky A , Östlund A, Nilsson T, Weichenhan D, Lutsik P, Bähr M, . Aberrant *MNX1* expression associated with t(7;12)(q36;p13) pediatric acute myeloid leukemia induces the disease through altering histone methylation. Haematologica 2024;109:725–39. 37317878 10.3324/haematol.2022.282255 PMC10905087

[66] Lovén J , Hoke HA, Lin CY, Lau A, Orlando DA, Vakoc CR, . Selective inhibition of tumor oncogenes by disruption of super-enhancers. Cell 2013;153:320–34. 23582323 10.1016/j.cell.2013.03.036 PMC3760967

[67] Whyte WA , Orlando DA, Hnisz D, Abraham BJ, Lin CY, Kagey MH, . Master transcription factors and mediator establish super-enhancers at key cell identity genes. Cell 2013;153:307–19. 23582322 10.1016/j.cell.2013.03.035 PMC3653129

[68] Ghandi M , Huang FW, Jané-Valbuena J, Kryukov GV, Lo CC, McDonald ER, . Next-generation characterization of the cancer cell line encyclopedia. Nature 2019;569:503–8. 31068700 10.1038/s41586-019-1186-3 PMC6697103

[69] Goyal A , Bauer J, Hey J, Papageorgiou DN, Stepanova E, Daskalakis M, . DNMT and HDAC inhibition induces immunogenic neoantigens from human endogenous retroviral element-derived transcripts. Nat Commun 2023;14:6731. 37872136 10.1038/s41467-023-42417-w PMC10593957

[70] Fenaux P , Gobbi M, Kropf PL, Issa J-PJ, Roboz GJ, Mayer J, . Guadecitabine vs treatment choice in newly diagnosed acute myeloid leukemia: a global phase 3 randomized study. Blood Adv 2023;7:5027–37. 37276510 10.1182/bloodadvances.2023010179 PMC10471926

[71] Chen X , Schulz-Trieglaff O, Shaw R, Barnes B, Schlesinger F, Källberg M, . Manta: rapid detection of structural variants and indels for germline and cancer sequencing applications. Bioinformatics 2016;32:1220–2. 26647377 10.1093/bioinformatics/btv710

[72] Boeva V , Popova T, Bleakley K, Chiche P, Cappo J, Schleiermacher G, . Control-FREEC: a tool for assessing copy number and allelic content using next-generation sequencing data. Bioinformatics 2012;28:423–5. 22155870 10.1093/bioinformatics/btr670 PMC3268243

[73] Cortés-Ciriano I , Lee JJ-K, Xi R, Jain D, Jung YL, Yang L, . Comprehensive analysis of chromothripsis in 2,658 human cancers using whole-genome sequencing. Nat Genet 2020;52:331–41. 32025003 10.1038/s41588-019-0576-7 PMC7058534

[74] Sollier E , Heilmann J, Gerhauser C, Scherer M, Plass C, Lutsik P. Figeno: multi-region genomic figures with long-read support. Bioinformatics 2024;40:btae354. 38857451 10.1093/bioinformatics/btae354 PMC11184262

[75] Dobin A , Davis CA, Schlesinger F, Drenkow J, Zaleski C, Jha S, . STAR: ultrafast universal RNA-seq aligner. Bioinformatics 2013;29:15–21. 23104886 10.1093/bioinformatics/bts635 PMC3530905

[76] Patro R , Duggal G, Love MI, Irizarry RA, Kingsford C. Salmon provides fast and bias-aware quantification of transcript expression. Nat Methods 2017;14:417–9. 28263959 10.1038/nmeth.4197 PMC5600148

[77] Love MI , Huber W, Anders S. Moderated estimation of fold change and dispersion for RNA-seq data with DESeq2. Genome Biol 2014;15:550. 25516281 10.1186/s13059-014-0550-8 PMC4302049

[78] Stephens M . False discovery rates: a new deal. Biostatistics 2017;18:275–94. 27756721 10.1093/biostatistics/kxw041 PMC5379932

[79] Ritchie ME , Phipson B, Wu D, Hu Y, Law CW, Shi W, . Limma powers differential expression analyses for RNA-sequencing and microarray studies. Nucleic Acids Res 2015;43:e47. 25605792 10.1093/nar/gkv007 PMC4402510

[80] Hao Y , Stuart T, Kowalski MH, Choudhary S, Hoffman P, Hartman A, . Dictionary learning for integrative, multimodal and scalable single-cell analysis. Nat Biotechnol 2024;42:293–304. 37231261 10.1038/s41587-023-01767-y PMC10928517

[81] Butler A , Hoffman P, Smibert P, Papalexi E, Satija R. Integrating single-cell transcriptomic data across different conditions, technologies, and species. Nat Biotechnol 2018;36:411–20. 29608179 10.1038/nbt.4096 PMC6700744

[82] Kiselev VY , Yiu A, Hemberg M. scmap: projection of single-cell RNA-seq data across data sets. Nat Methods 2018;15:359–62. 29608555 10.1038/nmeth.4644

[83] Gao T , Soldatov R, Sarkar H, Kurkiewicz A, Biederstedt E, Loh P-R, . Haplotype-aware analysis of somatic copy number variations from single-cell transcriptomes. Nat Biotechnol 2023;41:417–26. 36163550 10.1038/s41587-022-01468-y PMC10289836

[84] ENCODE Project Consortium . An integrated encyclopedia of DNA elements in the human genome. Nature 2012;489:57–74. 22955616 10.1038/nature11247 PMC3439153

[85] Zeller C , Richter D, Jurinovic V, Valtierra-Gutiérrez IA, Jayavelu AK, Mann M, . Adverse stem cell clones within a single patient’s tumor predict clinical outcome in AML patients. J Hematol Oncol 2022;15:25. 35279202 10.1186/s13045-022-01232-4 PMC8917742

[86] van de Werken HJG , Landan G, Holwerda SJB, Hoichman M, Klous P, Chachik R, . Robust 4C-seq data analysis to screen for regulatory DNA interactions. Nat Methods 2012;9:969–72. 22961246 10.1038/nmeth.2173

[87] Carter B , Ku WL, Kang JY, Hu G, Perrie J, Tang Q, . Mapping histone modifications in low cell number and single cells using antibody-guided chromatin tagmentation (ACT-seq). Nat Commun 2019;10:3747. 31431618 10.1038/s41467-019-11559-1 PMC6702168

[88] Skene PJ , Henikoff S. A simple method for generating high-resolution maps of genome-wide protein binding. Elife 2015;4:e09225. 26079792 10.7554/eLife.09225 PMC4480131

[89] Corces MR , Trevino AE, Hamilton EG, Greenside PG, Sinnott-Armstrong NA, Vesuna S, . An improved ATAC-seq protocol reduces background and enables interrogation of frozen tissues. Nat Methods 2017;14:959–62. 28846090 10.1038/nmeth.4396 PMC5623106

[90] Krijger PHL , Geeven G, Bianchi V, Hilvering CRE, de Laat W. 4C-seq from beginning to end: a detailed protocol for sample preparation and data analysis. Methods 2020;170:17–32. 31351925 10.1016/j.ymeth.2019.07.014

[91] Nilsson T , Waraky A, Östlund A, Li S, Staffas A, Asp J, . An induced pluripotent stem cell t(7;12)(q36;p13) acute myeloid leukemia model shows high expression of MNX1 and a block in differentiation of the erythroid and megakaryocytic lineages. Int J Cancer 2022;151:770–82. 35583991 10.1002/ijc.34122 PMC9545334

[92] Wermke M , Camgoz A, Paszkowski-Rogacz M, Thieme S, von Bonin M, Dahl A, . RNAi profiling of primary human AML cells identifies *ROCK1* as a therapeutic target and nominates fasudil as an antileukemic drug. Blood 2015;125:3760–8. 25931586 10.1182/blood-2014-07-590646

