# Supplementary material for: Enhancer Hijacking Discovery in Acute Myeloid Leukemia by Pyjacker Identifies MNX1 Activation via Deletion 7q
Source: Blood Cancer Discov. 2025 Mar 31;6(4):343–63. doi: 10.1158/2643-3230.BCD-24-0278 (PMC12209774; doi:10.1158/2643-3230.BCD-24-0278)
Supplement: Supplementary Figures — Supplementary Figure 1. Summary of the somatic alterations in the 39 ckAML samples. Supplementary Figure 2. Proportion of samples expressing the top pyjacker hits, for several AML cohorts profiled with RNA-seq. Supplementary Figure 3. Example rearrangements leading to gene activation and TP53 inactivation. Supplementary Figure 4. Rearrangements leading to MECOM monoallelic expression in sample 15KM20146. Supplementary Figure 5. Validation of the translocation t(1;3)(p36;q21) in sample 16KM11270 and of the breakpoints for the CDK6 enhancer duplication next to MNX1 in sample 15PB8708 by genomic PCR. Supplementary Figure 6. Deletion between the TCR beta locus and MNX1 leading to MNX1 expression. Supplementary Figure 7. Alternative rearrangements leading to MNX1 expression. Supplementary Figure 8. Differential gene expression analysis of MNX1 status in adult and pediatric AML. Supplementary Figure 9. Gene expressions of 25 selected cancer and hematological development associated genes differentially expressed under MNX1 activation. Supplementary Figure 10. Copy number alterations on chromosome 7 for samples profiled with scRNA-seq. Supplementary Figure 11. Reciprocal 4C. Supplementary Figure 12: Knockdown of MNX1 reduces tumor load of AML PDX cells in vivo. [file bcd-24-0278_supplementary_figures_suppsf1.pdf]

Supplementary Material

Pyjacker identifies enhancer hijacking events in acute myeloid leukemia including *MNX1* activation via deletion 7q

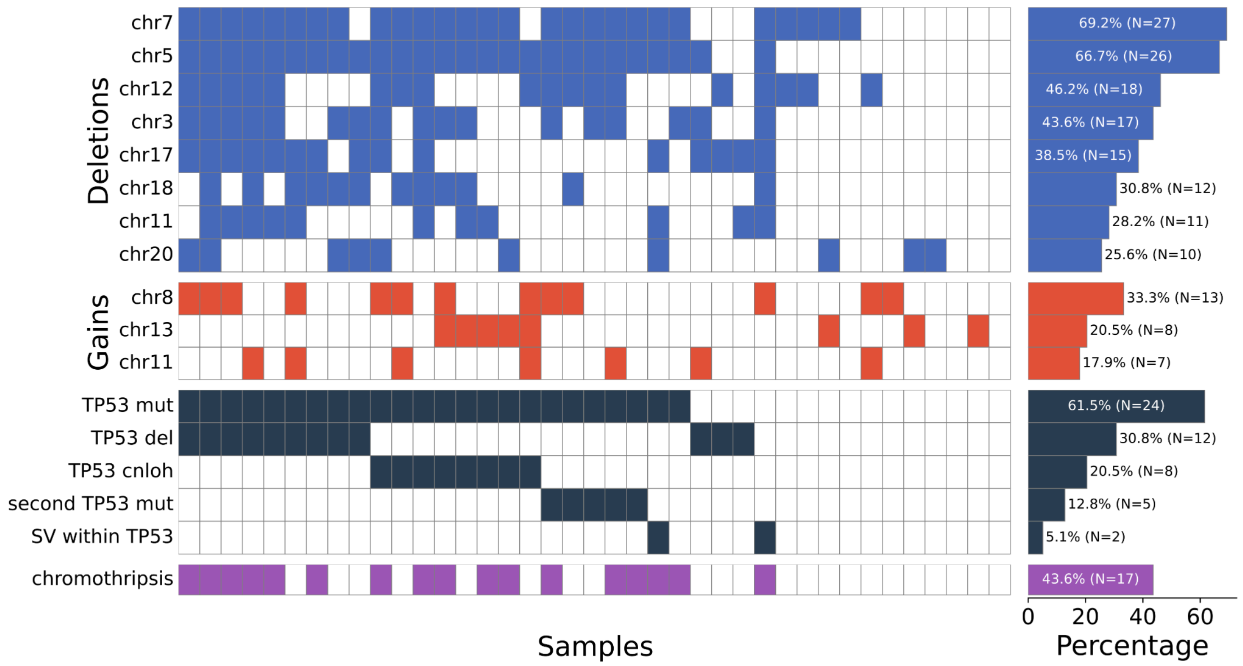

Supplementary Figure 1. Summary of the somatic alterations in the 39 ckAML samples. Heatmap showing the most common copy number alterations, TP53 status and chromothripsis status for the cohort of 39ckAML samples profiled with WGS and RNAseq. For copy number alterations, we counted each chromosome having at least 1Mb deleted or gained. Mut indicates mutation, del deletion, cnloh copy-neutral loss of heterozygosity, sv structural variant.

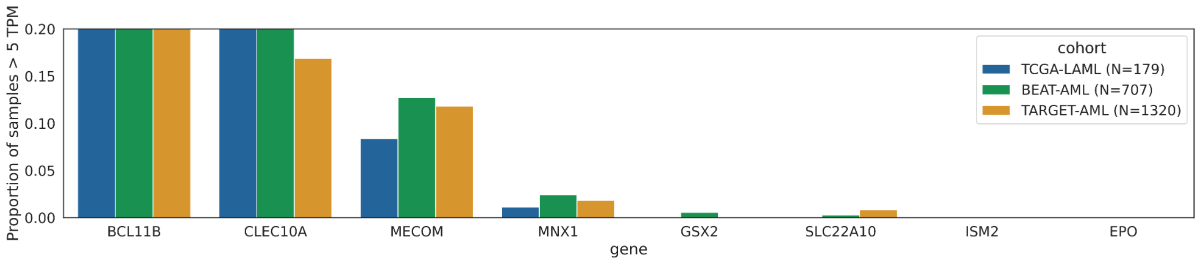

Supplementary Figure 2. Proportion of samples expressing the top pyjacker hits, for several AML cohorts profiled with RNA-seq. *BCL11B* and *CLEC10A* are expressed in normal T-cells and dendritic cells, respectively, which explains their expression in many samples.

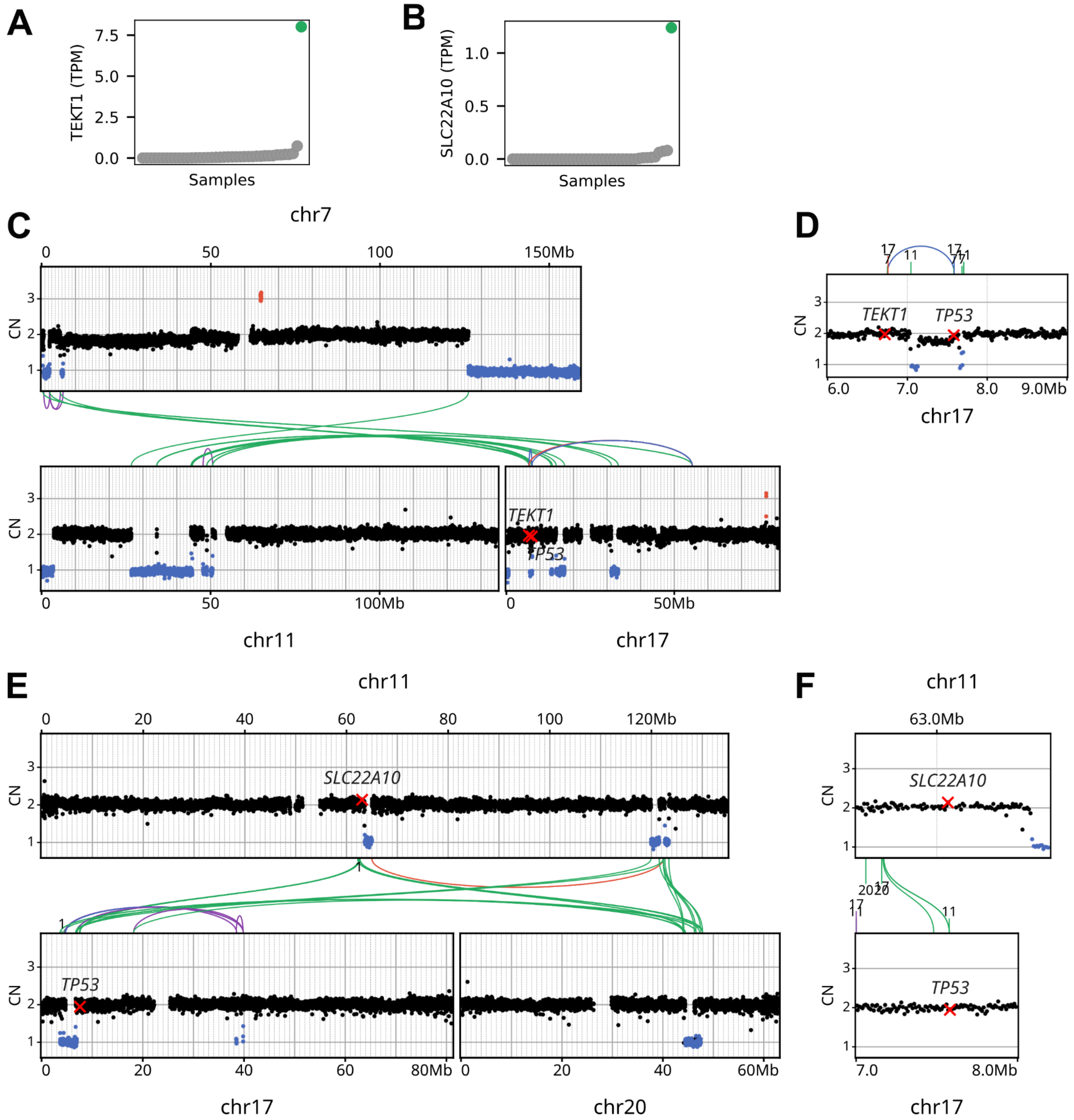

Supplementary Figure 3. Example rearrangements leading to gene activation and TP53 inactivation. A. *TEKT1* expression in transcript per million (TPM) in all samples, with sample 16PB3075 (with breakpoint close to *TEKT1*) highlighted in green. **B**. *SLC22A10* expression in all samples, with sample 15KM20146 (with breakpoint close to *SLC22A10*) highlighted in green. **C**. Copy numbers (CN) and SVs on chromosomes 7,11 and 17 in sample 16PB3075. Copy number losses are indicated in blue and gains in red. SVs are shown as arcs at the top, where the color indicates the orientation of the breakpoint: blue for deletion, red for duplication, and purple for inversion. **D**. Copy numbers and SVs around *TEKT1* and *TP53* in sample 16PB3075. **E**. Copy numbers and SVs on chromosomes 11,17 and 20 in sample 15KM20146. **F**. Copy numbers and SVs around *SLC22A10* and *TP53* in sample 15KM20146.

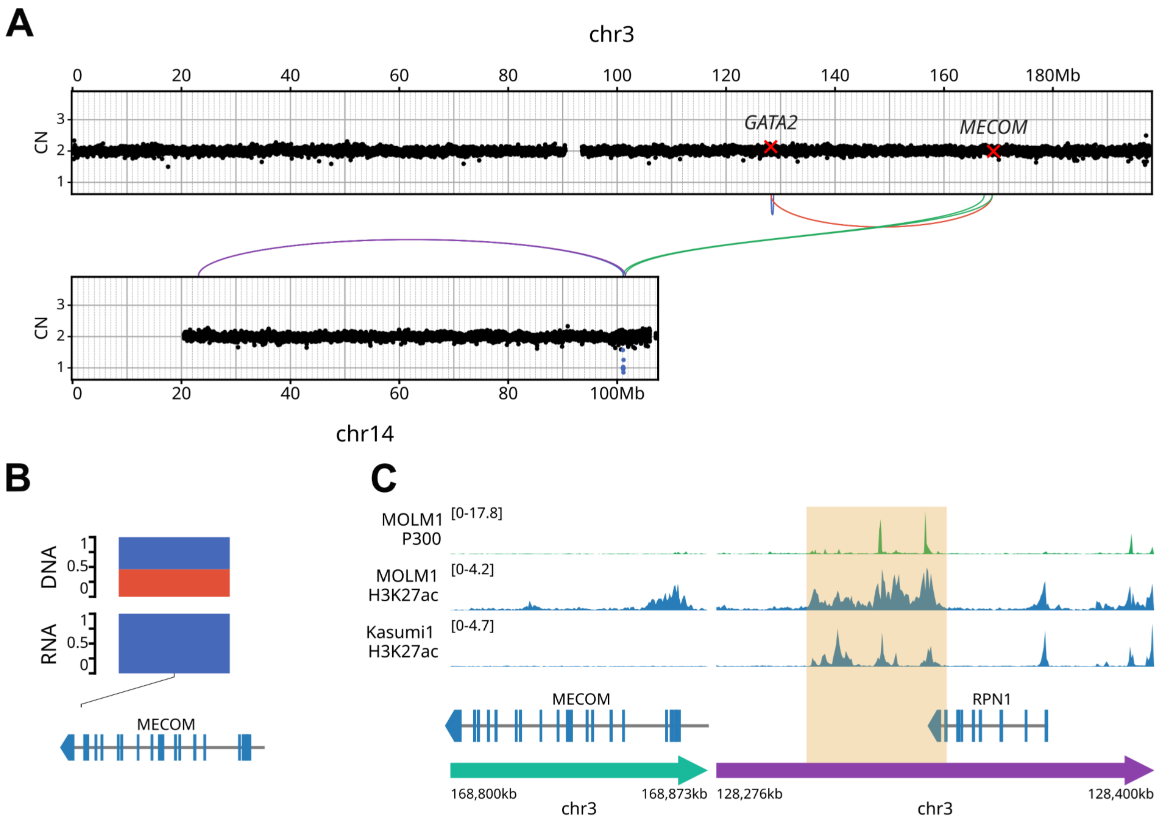

Supplementary Figure 4. Rearrangements leading to MECOM monoallelic expression in sample 15KM20146. **A**. Copy numbers (CN) and SVs on chr3 and chr14 for sample 15KM20146. Copy number losses are indicated in blue and gains in red. SVs are shown as arcs, where the color indicates the orientation of the breakpoint: blue for deletion, red for duplication, and purple for inversion. **B**. Variant allele frequencies of SNPs in *MECOM*, in DNA and RNA of sample 15KM20146 (major allele frequencies in blue and minor allele frequencies in red). **C**. H3K27ac and P300 tracks around the breakpoint leading to *MECOM* expression in sample 15KM20146. The putative enhancer is highlighted in orange.

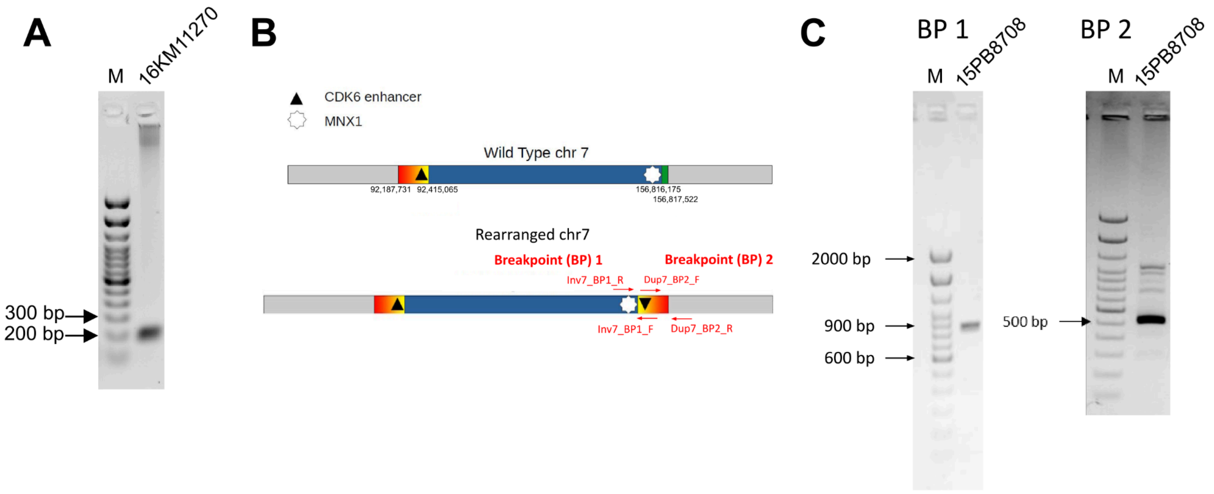

Supplementary Figure 5. Validation of the translocation t(1;3)(p36;q21) in sample 16KM11270 and of the breakpoints for the *CDK6* enhancer duplication next to *MNX1* in sample 15PB8708 by genomic PCR. A. PCR product of 227 bp for the t(1;3) translocation in sample 16KM11270 (chr1:2892507 to chr3:128244534). **B.** Schematic representation of the *CDK6* enhancer duplication next to *MNX1* in AML patient 15PB8708. The duplicated region is shown with a gradient color to show that it is inserted in inverse orientation. The region between the duplicated segment and the location where it is inserted is shown in blue. A small region shown in green is deleted at the location of the insertion. **C**. PCR product of 831 bp for the first breakpoint (BP1; chr7:156,816,175 to chr7:92,415,065), and PCR product of 500 bp for the second breakpoint (BP2; chr7:156,817,522 to chr7:92,187,731).

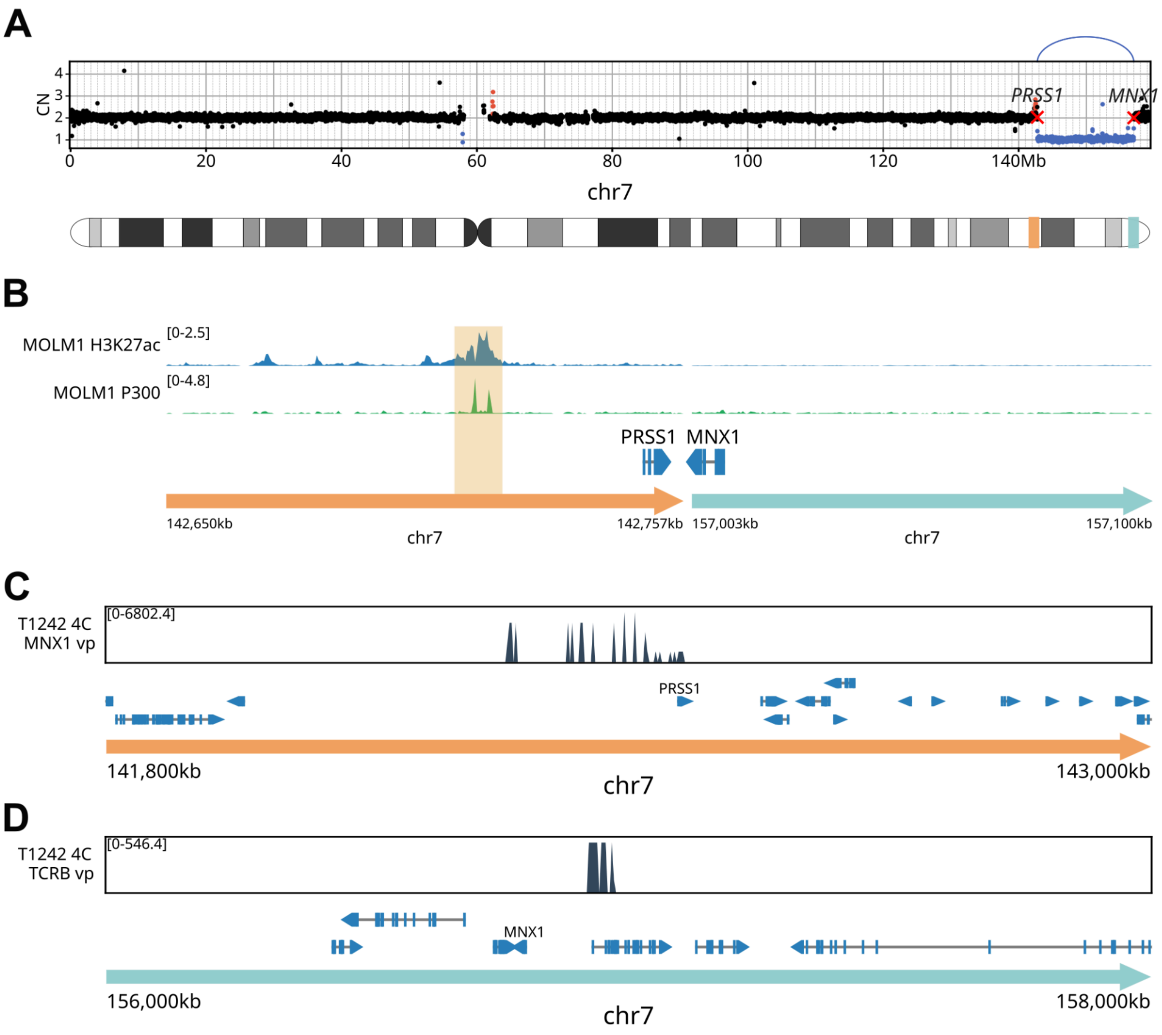

Supplementary Figure 6. Deletion between the TCR beta locus and *MNX1* leading to *MNX1* expression. A. Copy numbers (CN) and SVs on chr7 for sample T1242, with a smaller del(7q) leading to *MNX1* expression. Here, the coordinates are in hg38 reference because the left breakpoint (between *PRSS1* and *PRSS2*, within the T cell receptor beta locus) is in a region missing from the hg19 reference. **B**. ChIP-seq track of H3K27ac and P300 in MOLM-1 showing the putative enhancer responsible for *MNX1* activation in sample T1242 (hg38 reference). **C**. 4C track for sample T1242 with an *MNX1* viewpoint (vp), showing interaction with the TCR beta locus region (hg19 reference). **D**. 4C track for sample T1242 with a viewpoint at the TCR beta locus, showing interaction with the *MNX1* region (hg19 reference).

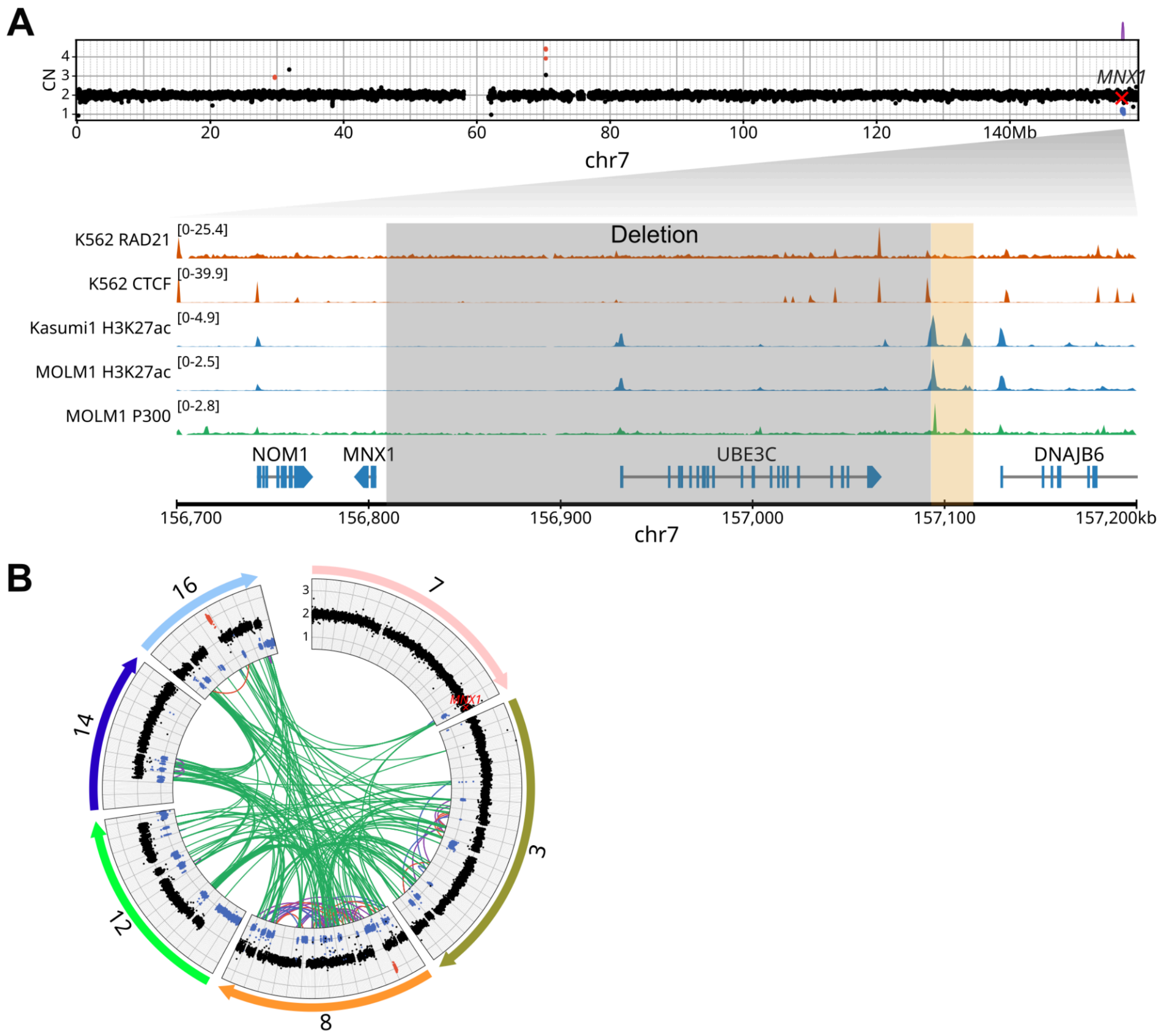

Supplementary Figure 7. Alternative rearrangements leading to *MNX1* expression. A. Copy numbers (CN) and SVs for sample T9058, with a 300 kb deletion to the right of *MNX1*, and ChIP-seq tracks showing the putative enhancer (highlighted in orange) to the right of the deleted region (highlighted in gray). **B**. Copy numbers and SVs in sample U4712, with a complex chromothripsis event involving multiple chromosomes, including breakpoints near *MNX1*.

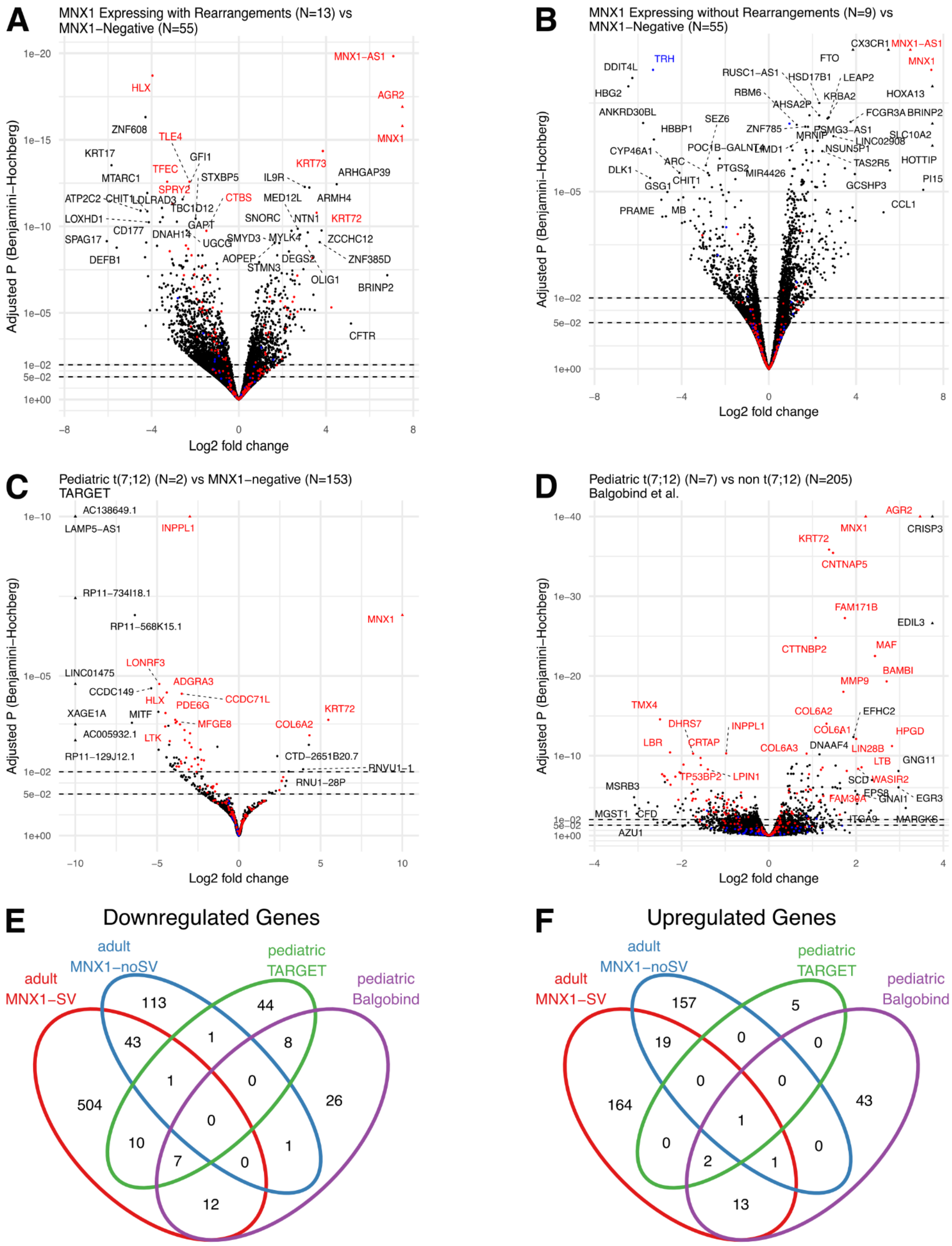

Supplementary Figure 8. Differential gene expression analysis of *MNX1* status in adult and pediatric AML. Genes marked in red have been proposed as an *MNX1*-associated gene signature in t(7;12) pediatric AML (PMID 36057683). Genes marked in blue have been proposed as an *NPM1*-associated gene signature (PMID 16109776). **A**. Volcano plot of adult AML with *MNX1* expression and an associated SV compared to *MNX1*-negative. **B**. Volcano plot of adult AML with *MNX1* expression without an associated SV compared to *MNX1*-negative. **C-D**. Volcano plots of pediatric AML with t(7;12)(q36;p13) compared to all other karyotypes, for two cohorts: microarray data from Balgobind et al. 2011 (**C**) and RNA-seq data from TARGET-AML (**D**). **E-F**. Intersection analysis of significantly downregulated (**E**) or upregulated (**F**) genes across the four comparisons (abs(-log10(Padj)*log(FC)>=5)).

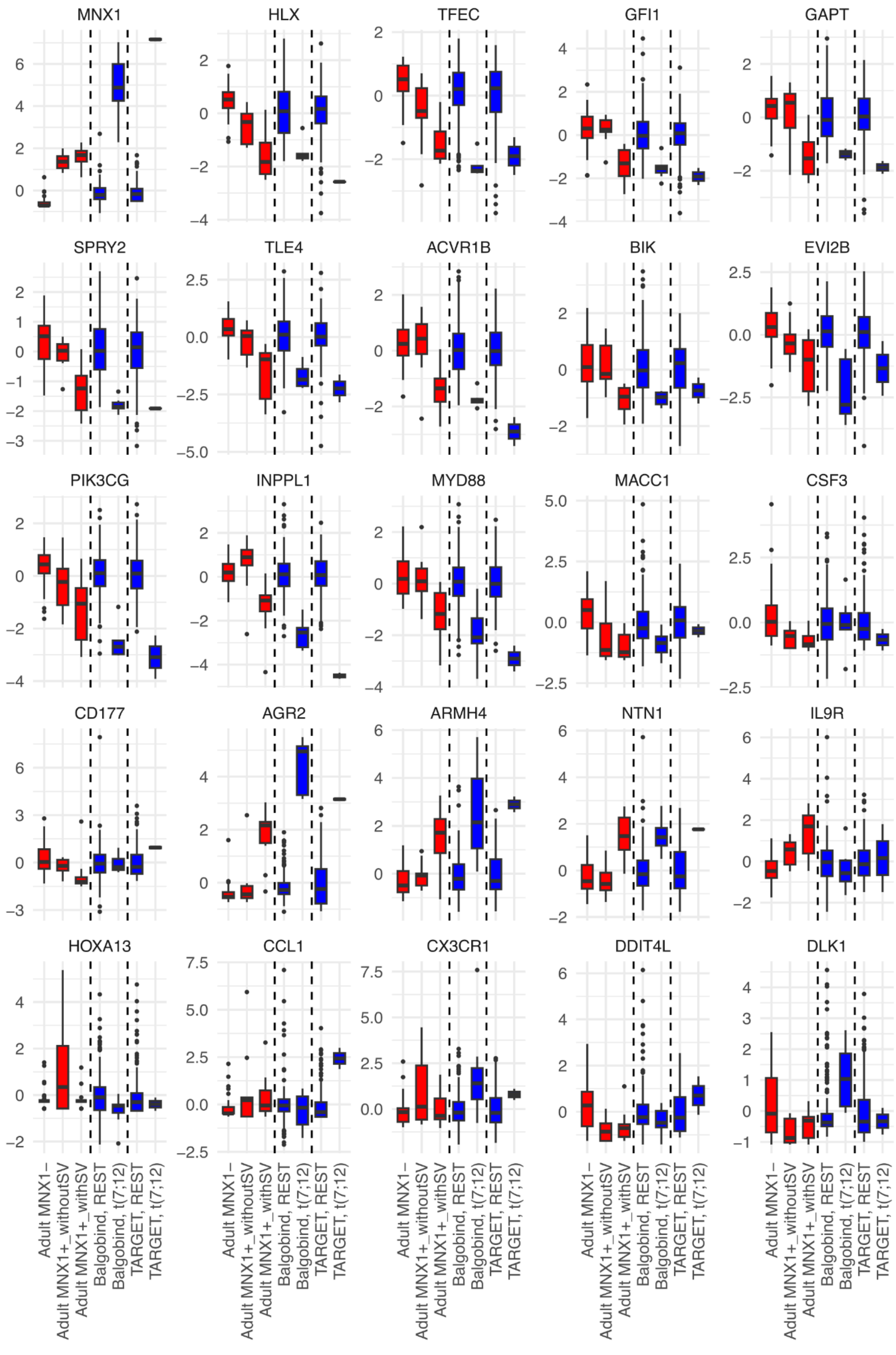

Supplementary Figure 9. Gene expressions of 25 selected cancer and hematological development associated genes differentially expressed under *MNX1* activation. Red boxplots represent the adult AML cohort presented in this study, whereas the blue boxplots represent the previously published Balgobind et al. and TARGET pediatric AML cohorts. The values are Z scores of vst-transformed normalized expressions. For the boxplots, the rectangles

extend from 25th to 75th percentiles, with a line indicating the median. Whiskers extend to 1.5 times the interquartile range, and outliers are shown individually.

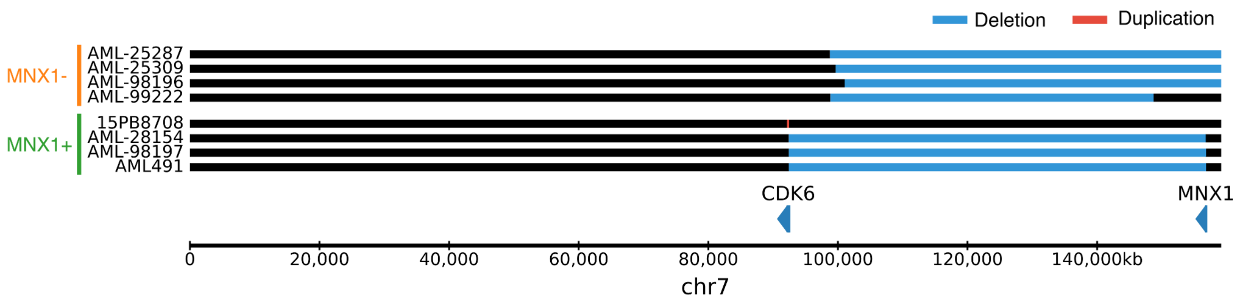

Supplementary Figure 10. **Copy number alterations on chromosome 7 for samples profiled with scRNA-seq.** Four *MNX1*-expressing samples (three with del(7q) and one with the *CDK6* enhancer duplicated next to *MNX1*) were profiled, as well as four control samples with del(7q) but with different breakpoints, not resulting in *MNX1* activation. Samples AML-25287, AML-98196, and AML-99222 are from bone marrow and samples 15PB8708, AML-25309, and AML-98197 are from peripheral blood. AML491 is a PDX sample.

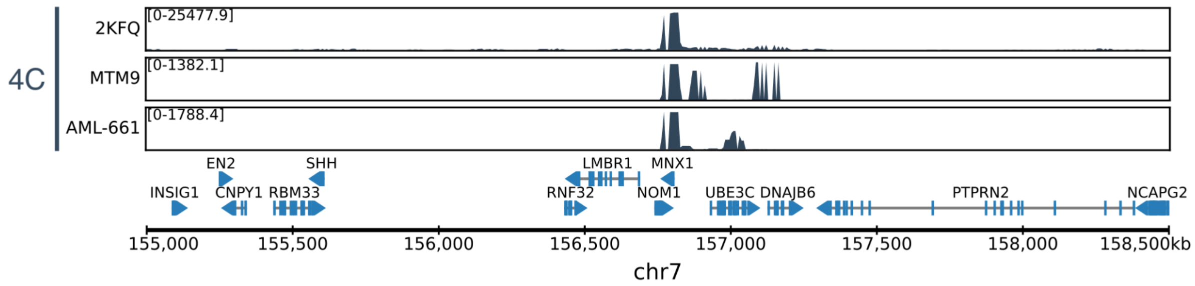

Supplementary Figure 11. **Reciprocal 4C.** 4C data using as viewpoint the *CDK6* region (chr7:92268000 hg19), shown in the region around *MNX1*, for del(7q) samples: MTM9 is an AML patient sample, AML-661 is a PDX sample derived from an AML patient with del(7q).

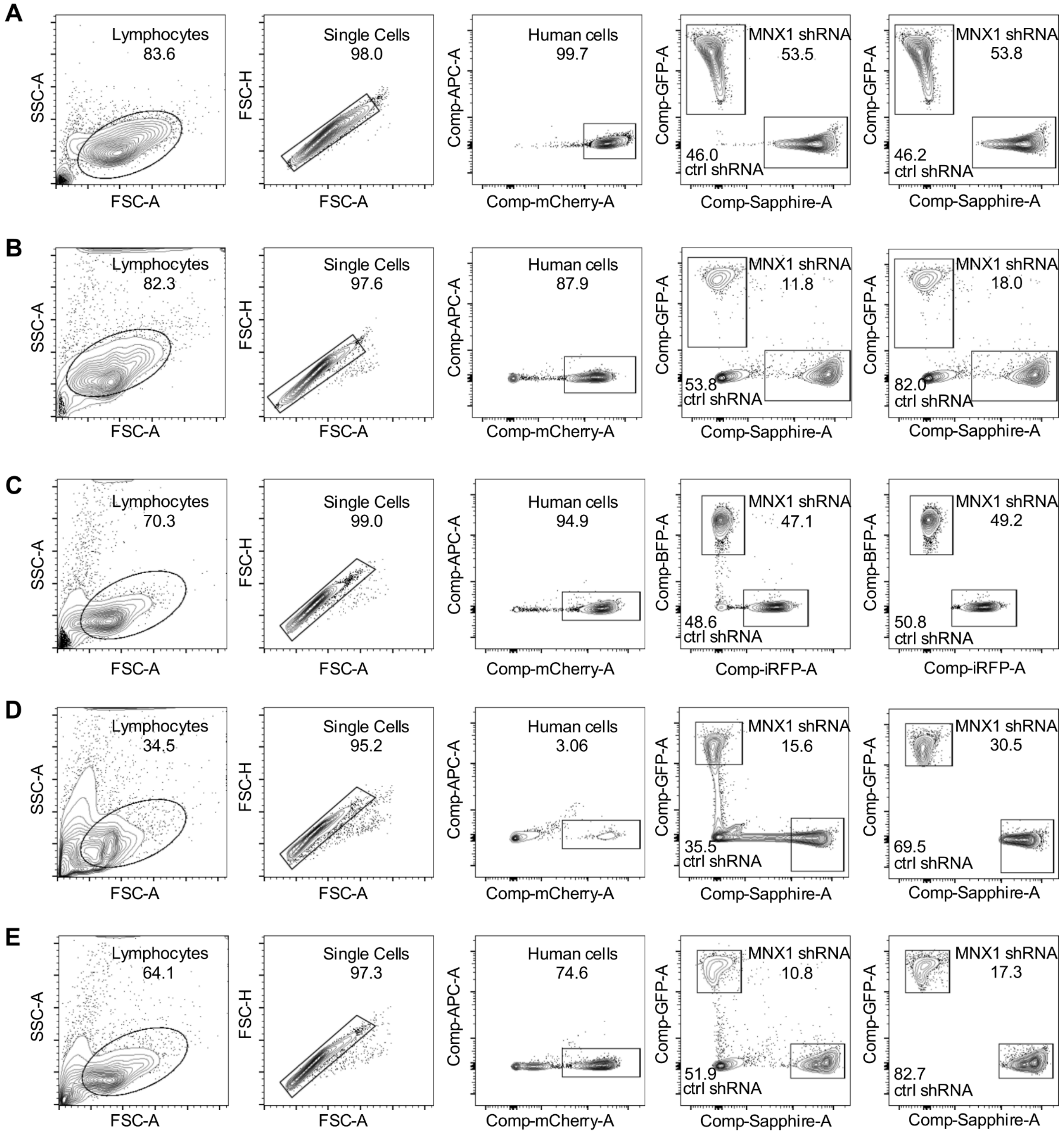

Supplementary Figure 12: Knockdown of *MNX1* reduces tumor load of AML PDX cells *in vivo*. Representative flow cytometry data and gating strategy related to Figure 7.

A-B. Representative raw flow cytometry data of Figure 7B of the input mixes (**A)** and the BM output samples (**B**) of *MNX1* shRNA and control AML-661 PDX cells. The following gating strategy was applied to all samples: Events are gated for lymphocytes and single cells by FSC and SSC. Human PDX cells are discriminated by mCherry fluorochrome expression. *MNX1* shRNA and control shRNA populations are measured by GFP or T-Sapphire, respectively, on mCherry positive cells and Boolean gates are applied for the final percentage values.

C-E. Representative raw flow cytometry data of **Figure 7D** of the input mixes (**C)**, the 3 days after TAM induction samples (**D**) and the BM output samples (**E**) of *MNX1* shRNA and control AML-661 PDX cells. The following gating strategy was applied to all samples: Events are gated for lymphocytes and single cells by FSC and SSC. Human PDX cells are discriminated by mCherry fluorochrome expression. *MNX1* shRNA and control shRNA populations before TAM induction are measured by BFP or iRFP, and *MNX1* shRNA and control shRNA populations after TAM induction are measured by GFP or T-Sapphire, respectively, on mCherry positive cells. Boolean gates are applied for the final percentage values.
